# Overcoming chemoresistance of small-cell lung cancer through stepwise HER2-targeted antibody-dependent cell-mediated cytotoxicity and VEGF-targeted antiangiogenesis

**DOI:** 10.1038/srep02669

**Published:** 2013-09-16

**Authors:** Toshiyuki Minami, Takashi Kijima, Satoshi Kohmo, Hisashi Arase, Yasushi Otani, Izumi Nagatomo, Ryo Takahashi, Kotaro Miyake, Masayoshi Higashiguchi, Osamu Morimura, Shoichi Ihara, Kazuyuki Tsujino, Haruhiko Hirata, Koji Inoue, Yoshito Takeda, Hiroshi Kida, Isao Tachibana, Atsushi Kumanogoh

**Affiliations:** 1 Department of Respiratory Medicine, Allergy and Rheumatic Diseases, Osaka University Graduate School of Medicine, Osaka, Japan; 2 Laboratory of Immunochemistry, World Premier International Research Center (WPI), Immunology Frontier Research Center, and Department of Immunochemistry, Research Institute for Microbial Disease, Osaka University, Suita, Osaka, Japan; 3 Core Research for Evolutional Science and Technology, Japan Science and Technology Agency, 4-1-8, Honcho Kawaguchi, Saitama 332-0012, Japan; 4 Department of Immunopathology, Immunology Frontier Research Center, Osaka University, Osaka, Japan

## Abstract

Small-cell lung cancer (SCLC) easily recurs with a multidrug resistant phenotype. However, standard therapeutic strategies for relapsed SCLC remain unestablished. We found that human epidermal growth factor receptor 2 (HER2) is not only expressed in pretreated human SCLC specimens, but is also upregulated when HER2-positive SCLC cells acquire chemoresistance. Trastuzumab induced differential levels of antibody-dependent cell-mediated cytotoxicity (ADCC) to HER2-positive SCLC cells. Furthermore, as a mechanism of the differential levels of ADCC, we have revealed that coexpression of intracellular adhesion molecule (ICAM)-1 on SCLC cells is essential to facilitate and accelerate the trastuzumab-mediated ADCC. Although SN-38–resistant SCLC cells lacking ICAM-1 expression were still refractory to trastuzumab, their *in vivo* growth was significantly suppressed by bevacizumab treatment due to dependence on their distinctive and abundant production of vascular endothelial growth factor. Collectively, stepwise treatment with trastuzumab and bevacizumab is promising for the treatment of chemoresistant SCLC.

Small-cell lung cancer (SCLC) accounts for approximately 15% of primary lung carcinomas and has the poorest outcome of all its histological types. The extreme aggressiveness of SCLC is due to its rapid doubling time, widespread metastases, and development of multidrug resistance (MDR) to chemotherapy^1^,^2^. The current front-line standard chemotherapy regimen for SCLC, cisplatin plus either etoposide or irinotecan, is effective in most SCLC cases, but the disease recurs with an MDR phenotype shortly after the initial treatment. In addition, there are currently no beneficial standard therapeutic strategies against the recurrent cancer^2^,^3^,^4^. Therefore, there is an urgent need to develop a novel strategy that overcomes MDR and confers significant survival benefits for patients. Although several clinical trials targeting receptor tyrosine kinases (RTKs) have been conducted for recurrent SCLC, they have yielded disappointing results^5^,^6^. The reasons for this inefficacy are that they are not the RTKs to which SCLC cells are dependent upon for their proliferation, and oncogenic driver mutations have not yet been found in SCLC^7^.

Human epidermal growth factor receptor 2 (HER2) belongs to the HER family of RTKs. HER2 can transduce cellular proliferative and survival signals either as a homodimer without ligand-stimulation or a heterodimer with other HER family members upon ligand stimulation^8^. HER2 is overexpressed in about 30% of breast cancers and its overexpression correlates with a poor outcome^9^,^10^. Similarly, HER2 expression is also an independent negative prognostic factor of extensive disease (ED)-SCLC^11^,^12^.

Trastuzumab, a humanized monoclonal antibody (Ab) against HER2, has already been approved for the treatment of HER2-overexpressing breast and gastric cancers^13^,^14^. Several mechanisms are proposed for the antitumor activity of trastuzumab, including inhibition of HER2-mediated signaling and antibody-dependent cell-mediated cytotoxicity (ADCC), which is exerted through natural killer (NK) cell–initiated cancer cell lysis^15^,^16^. SCLC cells have been shown to be generally susceptible to NK cell–mediated lysis^17^. Moreover, chemoresistant SCLC cells exhibit increased susceptibility to lymphokine-activated killer cells compared to their chemosensitive counterparts^18^. Based on these observations, we presumed that HER2 is targetable by trastuzumab especially in chemoresistant HER2-positive SCLC.

We here investigated the therapeutic potential and mechanisms of trastuzumab toward HER2-positive MDR SCLC. Furthermore, we evaluated the salvage therapeutic efficacy of bevacizumab, a humanized monoclonal Ab against vascular endothelial growth factor (VEGF), on trastuzumab-refractory SCLC.

## Results

### Establishment of a highly sensitive immunohistochemistry (IHC) system to detect HER2 in SCLC

**Figure 1:**
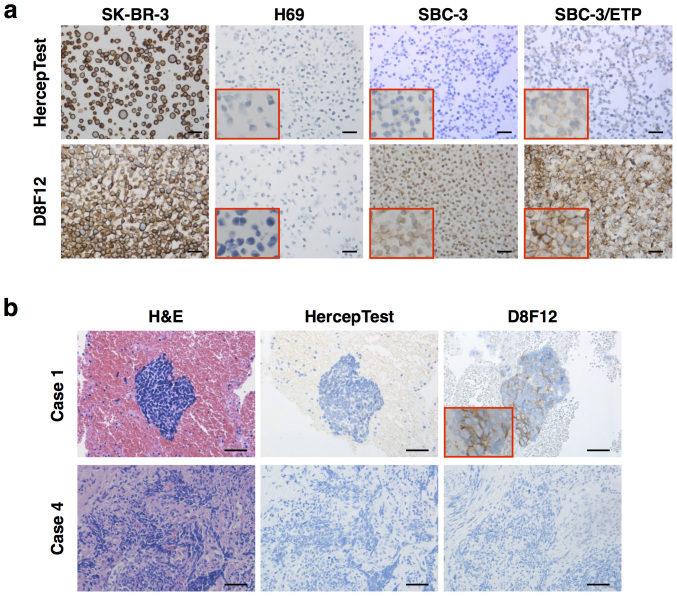
Development of a highly sensitive IHC system to detect HER2 in SCLC. (a) Detection of HER2 by IHC in breast cancer (SK-BR-3) and SCLC (H69, SBC-3, and SBC-3/ETP) cell blocks by HercepTest (top panels) or the new system (bottom panels). SK-BR-3 and H69 cells were used as a positive and negative control, respectively. The D8F12 Ab-based new IHC system exhibited improved sensitivity compared with HercepTest without compromising specificity. (b) The new IHC system also works well in human SCLC samples obtained by diagnostic biopsy. Representative histological images of HER2-positive (top panels) and -negative (bottom panels) cases are shown. All scale bars, 50 μm.

Since HER2 expression is upregulated in chemoresistant SCLC cells^19^, it is reasonable to target HER2 in SCLC patients who have become resistant to the front-line chemotherapy. HercepTest is commonly used to select eligible patients for trastuzumab therapy in breast and gastric cancer^14^,^20^,^21^. We first performed HercepTest using formalin-fixed paraffin-embedded blocks of SK-BR-3 (positive control, breast cancer cell line), H69 (negative control), SBC-3, and etoposide-resistant SBC-3/ETP cells. HER2 was strongly stained in SK-BR-3 cells, but not in parental SBC-3 cells and was faintly detected even in HER2-upregulated SBC-3/ETP cells (Figure 1a). These results led us to establish a new IHC detection system with higher sensitivity applicable to SCLC.

We performed the antigen retrieval step under higher alkaline conditions at pH 9.0, compared to the generally approved pH 6.0, to enhance the detection of cell surface membrane proteins. We also used a rabbit anti-human HER2 monoclonal Ab (clone D8F12) as the detection antibody. These two IHC methodological modifications succeeded in detecting HER2 in SBC-3 and SBC-3/ETP cells, while H69 cells remained HER2-negative, indicating that these modifications increased the sensitivity without compromising the specificity of the assay (Figure 1a). Moreover, we confirmed that this IHC system could also detect HER2 in human SCLC biopsy samples (Figure 1b). Of 10 samples obtained from individual patients, three were HER2-positive (Figure 1b and Supplementary Figure 1a and b), whereas nine were not stained at all shown as the representative two cases (Figure 1b) and only one specimen was faintly stained by HercepTest. Therefore, all cases were considered HER2-negative by HercepTest scoring system^21^.

### Effect of trastuzumab monotherapy against HER2-positive SCLC cells

To confirm whether trastuzumab can directly bind to cell surface HER2 on SCLC cells, we performed fluorescence-activated cell sorting (FACS) analysis using trastuzumab as the primary antibody. Trastuzumab recognized HER2 in six of 10 SCLC cell lines of Japanese origin but in none of three cell lines of Caucasian origin (Supplementary Figure 2a), suggesting the existence of ethnic difference about HER2 expression in SCLC. Moreover, it also detected HER2 upregulation in various types of chemoresistant SBC-3 sublines regardless of the differential resistant mechanisms (Supplementary Figure 2b).

**Figure 2:**
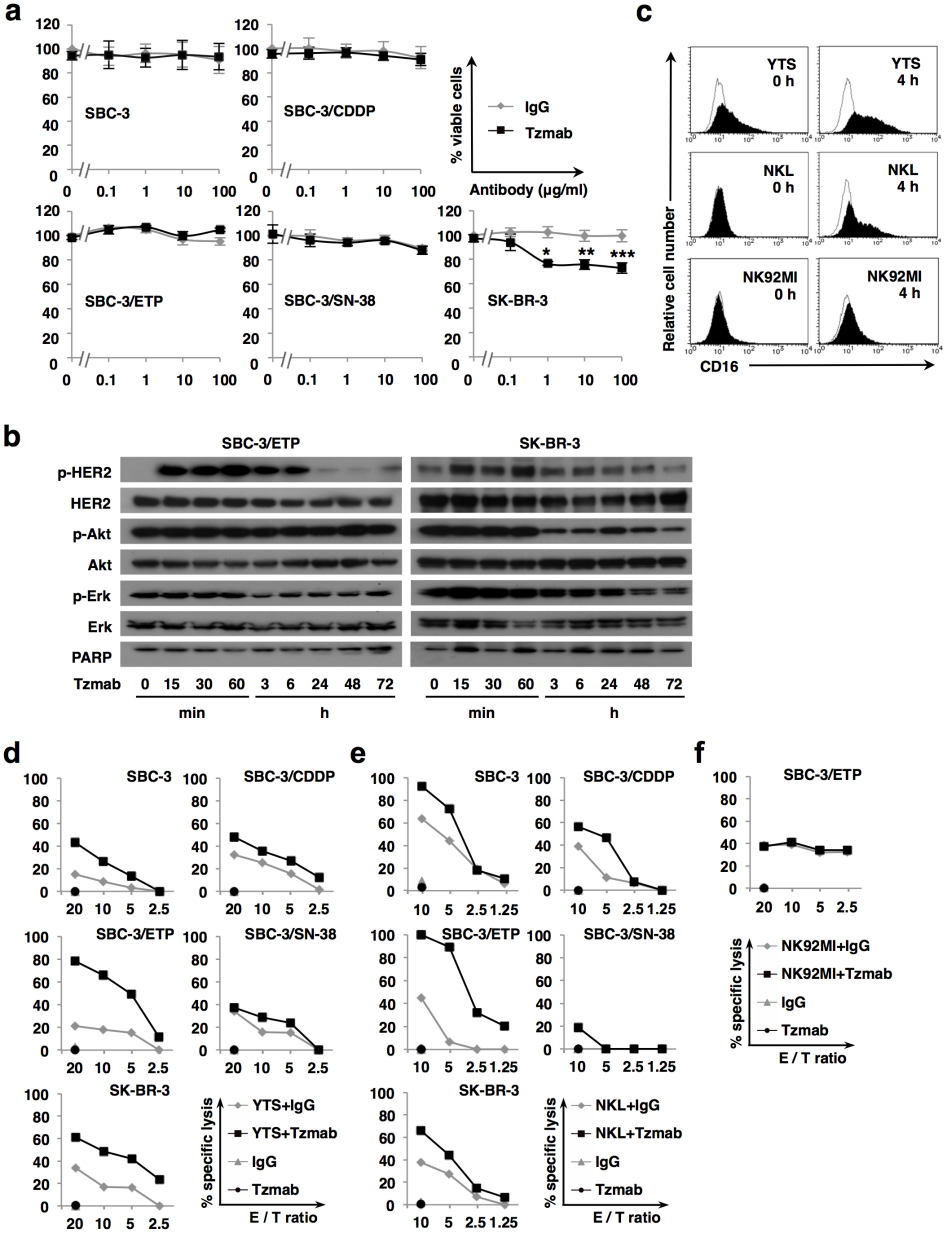
Antitumor activity of trastuzumab does not depend on direct inhibition of HER2 signaling but rather on ADCC in HER2-positive SCLC cells. (a) HER2-positive SCLC cells (SBC-3, SBC-3/CDDP, SBC-3/ETP, and SBC-3/SN-38 cells) and HER2-overexpressing breast carcinoma cells (SK-BR-3 cells) were treated with 10 μg/ml of normal human IgG or trastuzumab (Tzmab) for 72 h. The relative numbers of viable cells were quantified using the CCK-8 assay. Points, mean% viable cells; bars, SD of at least three independent experiments performed in triplicate; *, *P* = 0.018; **, *P* = 0.004; ***, *P* = 0.002. (b) SBC-3/ETP and SK-BR-3 cells were treated with 10 μg/ml of trastuzumab for up to 72 h. Phosphorylation and expression of HER2, Akt, and Erk in whole cell lysates were examined by immunoblotting. PARP was also examined to detect apoptosis. Each lane of SBC-3/ETP and SK-BR-3 cell lysates contains 60 μg and 30 μg of total protein, respectively. Representative blots from three independent experiments with similar results are shown. Blots are cropped in the figure and full-length blots are presented in Supplementary information. (c) Induction of CD16 expression on NK cells. NK cells (YTS, NKL, and NK92MI cells) were treated with or without 10 μg/ml of trastuzumab for 4 h. Then, the cells were labeled with an anti-CD16 monoclonal Ab (black shaded) or isotype-matched control (solid line) and analyzed for cell surface expression of CD16 by FACS. (d), (e), and (f) Evaluation of trastuzumab-mediated ADCC. Target cancer cells and effector NK cells were coincubated at various E/T ratios with 10 μg/ml of normal human IgG or trastuzumab (Tzmab) for 4 h. Cytotoxic activity was determined based on the LDH release assay. Representative data from at least three experiments are shown as the means of triplicate cultures.

We next investigated whether trastuzumab monotherapy could inhibit proliferation of HER2-positive SCLC cells. Various concentrations of either trastuzumab or human normal IgG ranging from 0 to 100 μg/ml were added to cultures of HER2-positive SCLC and breast cancer cells, and the antiproliferative effects were assessed by CCK-8, a tetrazolium reagent, assay. More than 1 μg/ml of trastuzumab significantly inhibited the proliferation of HER2-overexpressing SK-BR-3 cells but had no effect on HER2-positive SCLC cells (Figure 2a).

We then examined how trastuzumab affects HER2-mediated downstream signaling by comparing SBC-3/ETP cells with SK-BR-3 cells. Exposure to 10 μg/ml of trastuzumab increased the levels of phosphorylated HER2 in both SBC-3/ETP and SK-BR-3 cells for up to 6 h. However, the phosphorylation levels returned to basal levels after 24 h in both cell lines. Phosphorylations of Akt and extracellular signal–regulated kinase (Erk) 1/2 were remarkably decreased in SK-BR-3 cells from 3 to 72 h after trastuzumab exposure, whereas they were not affected throughout the 72-h treatment in SBC-3/ETP cells. Cleavage of poly (ADP-ribose) polymerase (PARP) was not observed in either cell line (Figure 2b). These results indicate that trastuzumab transiently upregulates the phosphorylation status of HER2 but does not affect the downstream mitogen-activated protein kinase and Akt pathways in SBC-3/ETP cells. This may explain why trastuzumab did not inhibit the proliferation of HER2-positive SCLC cells.

### Trastuzumab-mediated ADCC against HER2-positive SCLC cells

ADCC, as opposed to direct HER2-signaling inhibition, has been proposed as the major antitumor mechanism for trastuzumab^15^,^16^. We then tested whether trastuzumab could mediate ADCC against HER2-positive SCLC cells by coincubation assay with NK cells. We utilized 3 NK cell lines, YTS, NKL, and NK92MI. Before performing the ADCC assay, we examined the expression levels of Fcγ receptor III (CD16) on each NK cell line because this receptor is indispensable for ADCC. After 4-h exposure to trastuzumab, FACS analysis revealed that CD16 expression was upregulated in YTS and NKL cells but not in NK92MI cells (Figure 2c). Trastuzumab exerted various degrees of killing against HER2-positive SCLC and SK-BR-3 cells when cocultured with YTS or NKL cells. Notably, SBC-3/ETP cells were the most susceptible among the HER2-positive SCLC cells (Figure 2d and e). However, trastuzumab-mediated killing was not induced by NK92MI cells (Figure 2f). These results suggest that HER2-positive SCLC cells were lysed by trastuzumab-mediated ADCC.

### Antitumor effects of trastuzumab upon HER2-positive SCLC tumor xenografts

**Figure 3:**
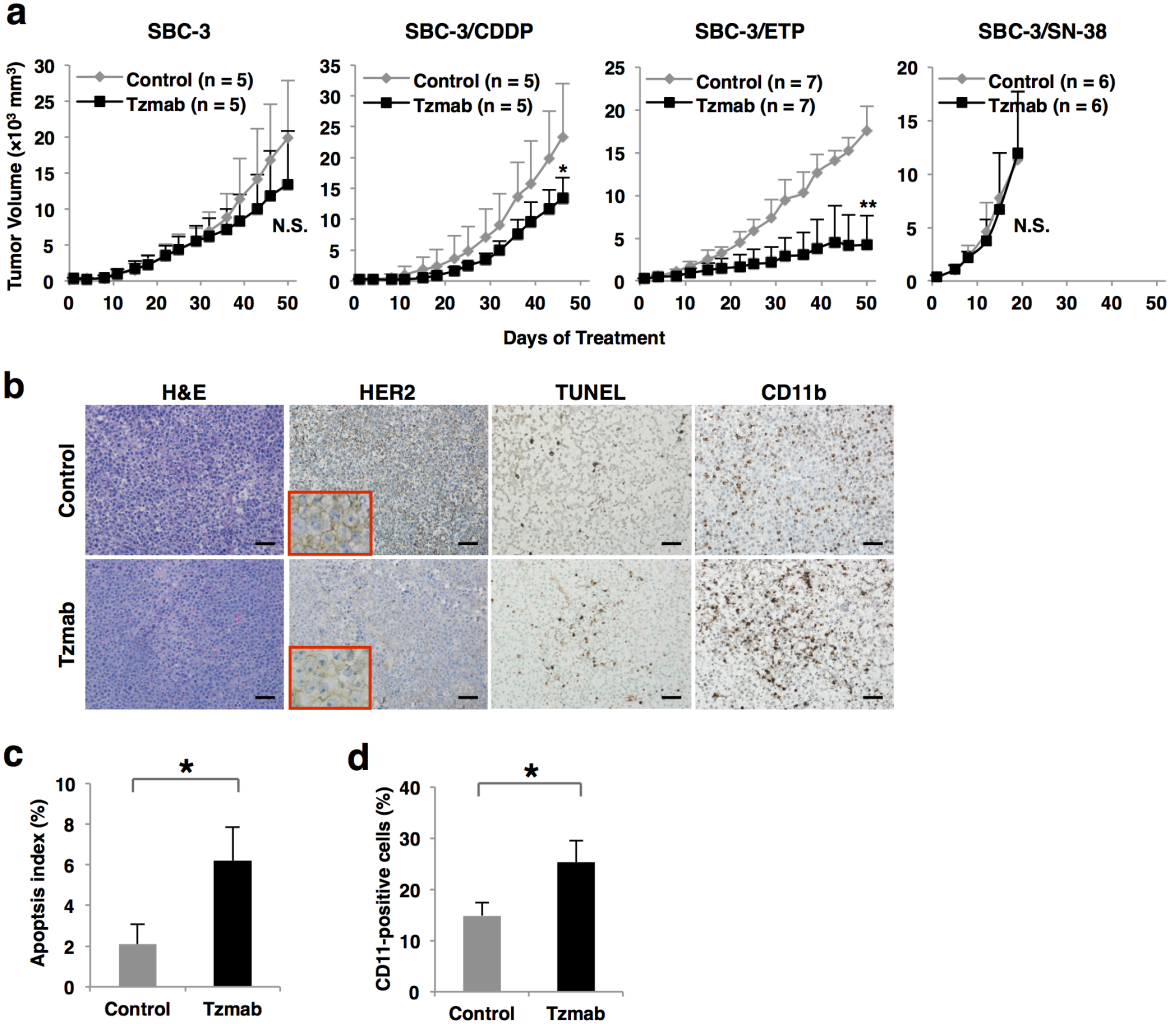
Antitumor effects of trastuzumab on HER2-positive SCLC tumor xenografts. (a) HER2-positive SCLC cells were inoculated subcutaneously into the flanks of athymic nude mice. When the tumor volume reached approximately 200–300 mm^3^, the mice were randomly assigned to the control (PBS) arm or trastuzumab (Tzmab) treatment arm (n = 5–7 mice per group). Trastuzumab was intraperitoneally administered at a dose of 30 mg/kg twice weekly. Points, mean tumor volumes; bars, SD; *, *P* = 0.042; **, *P* < 0.001. (b) Representative histological images of H&E and IHC for HER2, TUNEL, and CD11b. Scale bar, 50 μm. (c) Apoptosis index (%) was determined by calculating the number of TUNEL-positive cells per total number of cells, which consisted of 1000 or more SCLC cells in five randomly selected fields. *, *P* = 0.003. (d) CD11b-positive cells were quantified by counting at least 1000 cells in five randomly selected fields. *, *P* = 0.003.

We next investigated the antitumor efficacy of trastuzumab in nude mice bearing HER2-positive SCLC xenografts. As shown in Figure 3a, intraperitoneal injection of 30 mg/kg of trastuzumab twice weekly significantly inhibited the growth of cisplatin-resistant SBC-3/CDDP and SBC-3/ETP but not irinotecan-resistant SBC-3/SN-38 subcutaneous tumors in mice (*P* = 0.042 in SBC-3/CDDP xenografts and *P* < 0.001 in SBC-3/ETP xenografts). These results were consistent with the *in vitro* ADCC assays. Especially, tumor growth was inhibited in all of the seven trastuzumab-treated SBC-3/ETP tumor-bearing mice, two of which achieved complete remission. Additionaly, same tendency was confirmed at a lower dose administration of trastuzumab (10 mg/kg, twice weekly) (Supplementary Figure 3). Furthermore, in SBC-3/ETP xenografts, terminal deoxynucleotidyl transferase dUTP nick end labeling (TUNEL) staining showed that trastuzumab significantly induced apoptosis of SCLC cells (apoptosis index was 6.20 ± 1.63% in trastuzumab group *vs*. 2.10 ± 0.97% in control group, *P* = 0.003), despite the downregulation of cell surface HER2 expression upon trastuzumab treatment (Figure 3b and c). In addition, CD11b-positive tumor-infiltrating NK cells, macrophages, and granulocytes were significantly recruited into tumor foci upon trastuzumab treatment (25.3 ± 4.2% *vs*. 14.8 ± 2.6%, *P* = 0.003) (Figure 3b and d). By contrast, there was no significant increase in TUNEL- and CD11b-positive cells in SBC-3 xenografts upon trastuzumab treatment (Supplementary Figure 4a–c). These findings suggest that the *in vivo* antitumor effects of trastuzumab are accompanied by tumor-infiltrating ADCC-inducible CD11b-positive immune cells such as NK cells, macrophages and granulocyte.

### Internalization, ubuiquitination and lysosomal degradation of trastuzumab

**Figure 4:**
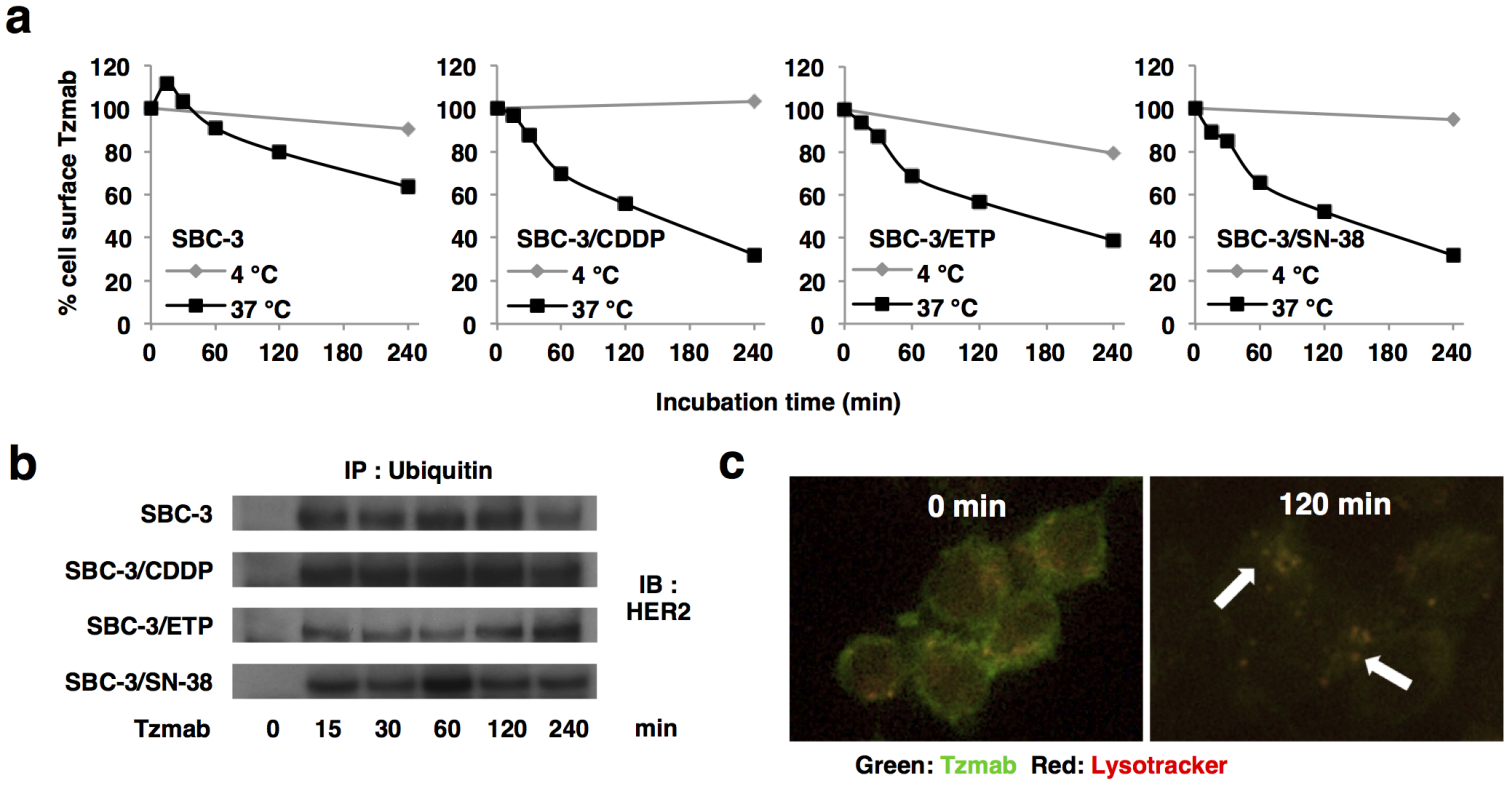
Trastuzumab is rapidly internalized, and subsequently undergoes ubiquitination and lysosomal degradation. (a) After HER2-positive SCLC cells were incubated with 10 μg/ml of trastuzumab for 45 min at 4°C, cells were warmed to 37°C to allow internalization or maintained at 4°C. The residual levels of cell surface trastuzumab were calculated based on the mean fluorescence intensity (MFI) analyzed by FACSort. (b) After HER2-positive SCLC cells were treated with 10 μg/ml of trastuzumab (Tzmab) for up to 240 min, whole cell lysates were immunoprecipitated with an Ab against ubiquitin. The immunoprecipitant was immunoblotted using an Ab against HER2 to detect HER2 ubiquitination. Blots are cropped in the figure and full-length blots are presented in Supplementary information. (c) SBC-3/CDDP cells were treated with 10 μg/ml of Alexa 488–labeled trastuzumab (green) in the presence of LysoTracker for 45 min at 4°C. Thereafter, trastuzumab was allowed to internalize for up to 120 min at 37°C and fluorescence images are shown. Arrows, lysosomal localization of trastuzumab.

To determine whether the difference of *in vivo* antitumor effects among xenografts depended on the difference of cell surface detention time and/or degradation time of trastuzumab-HER2 complexes, we performed internalization, ubuiquitination and lysosomal degradation assays. Unexpectedly, in all HER2-positive SCLC cells, the cell surface-binding trastuzumab diminished with time during the 37°C incubation, while most trastuzumab remained on cell surface at 4°C (Figure 4a). Moreover, HER2 ubiquitination had already started after 15 min of trastuzumab treatment (Figure 4b). Immunofluorescence studies also showed that most trastuzumab was internalized from the cell surface, colocalized with lysosomes, and underwent clustering after 120 min at 37°C (Figure 4c). From these observations, trastuzumab is internalized shortly after binding to HER2, subsequently undergoes ubiquitinqtion and lysosomal lysis. Therefore, we presumed that trastuzumab-mediated ADCC must occur rapidly after HER2 recognition.

### Intracellular adhesion molecule (ICAM)-1 as a facilitator and accelerator of trastuzumab-mediated ADCC

**Figure 5:**
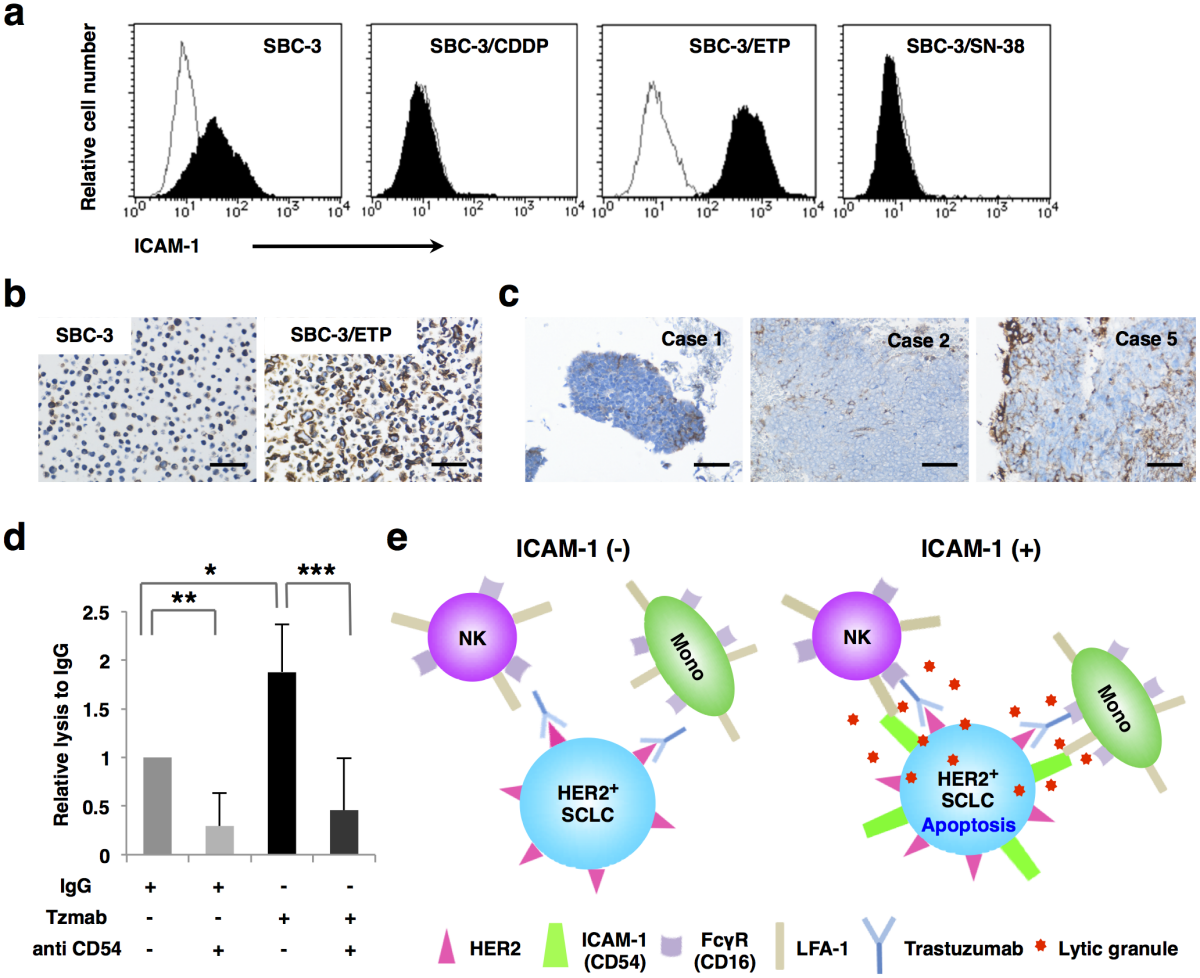
CAM-1 facilitates trastuzumab-mediated ADCC. (a) Parental and chemoresistant SBC-3 cells are labeled with 5 μg/ml of either an anti-CD54 mouse monoclonal Ab (black shaded) or an isotype-matched control Ab (solid line) and presented as histograms. (b) Upregulation of ICAM-1 expression on SBC-3/ETP cells compared to SBC-3/ETP cells was also confirmed by IHC. Scale bar, 50 μm. (c) SCLC cells in biopsy specimens from chemonaïve patients heterogeneously expressing ICAM-1. Scale bar, 50 μm. (d) SBC-3/ETP cells were coincubated with YTS cells in the presence of 10 μg/ml of normal human IgG or trastuzumab (Tzmab) with or without 5 μg/ml of an anti-human ICAM-1 (CD54) Ab. Column, mean relative lysis ratio compared to that of normal IgG; bars, SD of three independent experiments; *, *P* = 0.038; **, *P* = 0.026; ***, *P* = 0.025. (e) Facilitation of trastuzumab-mediated ADCC by ICAM-1 is schematized. NK, natural killer cell; Mono, macrophage and monocyte; FcγR, Fcγ receptor.

As a strong facilitator and accelerator of rapid ADCC completion, we considered the involvement of cell adhesion molecule to be crucial. Since all of the NK cells used as effectors in ADCC assay abundantly express lymphocyte function–associated antigen (LFA)-1, a heterodimer of CD11a and CD18 (Supplementary Figure 5), we evaluated the expression of its counter receptor, ICAM-1, on target SCLC cells. FACS and IHC analyses showed that ICAM-1 expression was remarkably upregulated in SBC-3/ETP cells compared to parental SBC-3 cells (Figure 5a and b). The same tendency was observed in other etoposide-resistant and parental SCLC cells (H69/VP and H69 cells) (Supplementary Figure 6). A population of chemonaïve SCLC cells in biopsy specimens was also positive for ICAM-1, although the expression pattern was heterogeneous (Figure 5c). Moreover, trastuzumab-mediated ADCC in SBC-3/ETP cells was significantly weakened in the presence of an ICAM-1–blocking antibody (Figure 5d). These results confirmed that trastuzumab-mediated ADCC is augmented by cell surface ICAM-1 expression in target SCLC cells. The supposed role of ICAM-1 involved in the mechanisms of trastuzumab-mediated ADCC is schematized in Figure 5e.

### Antitumor activity of bevacizumab against trastuzumab-refractory SBC-3/SN-38 xenografts

**Figure 6:**
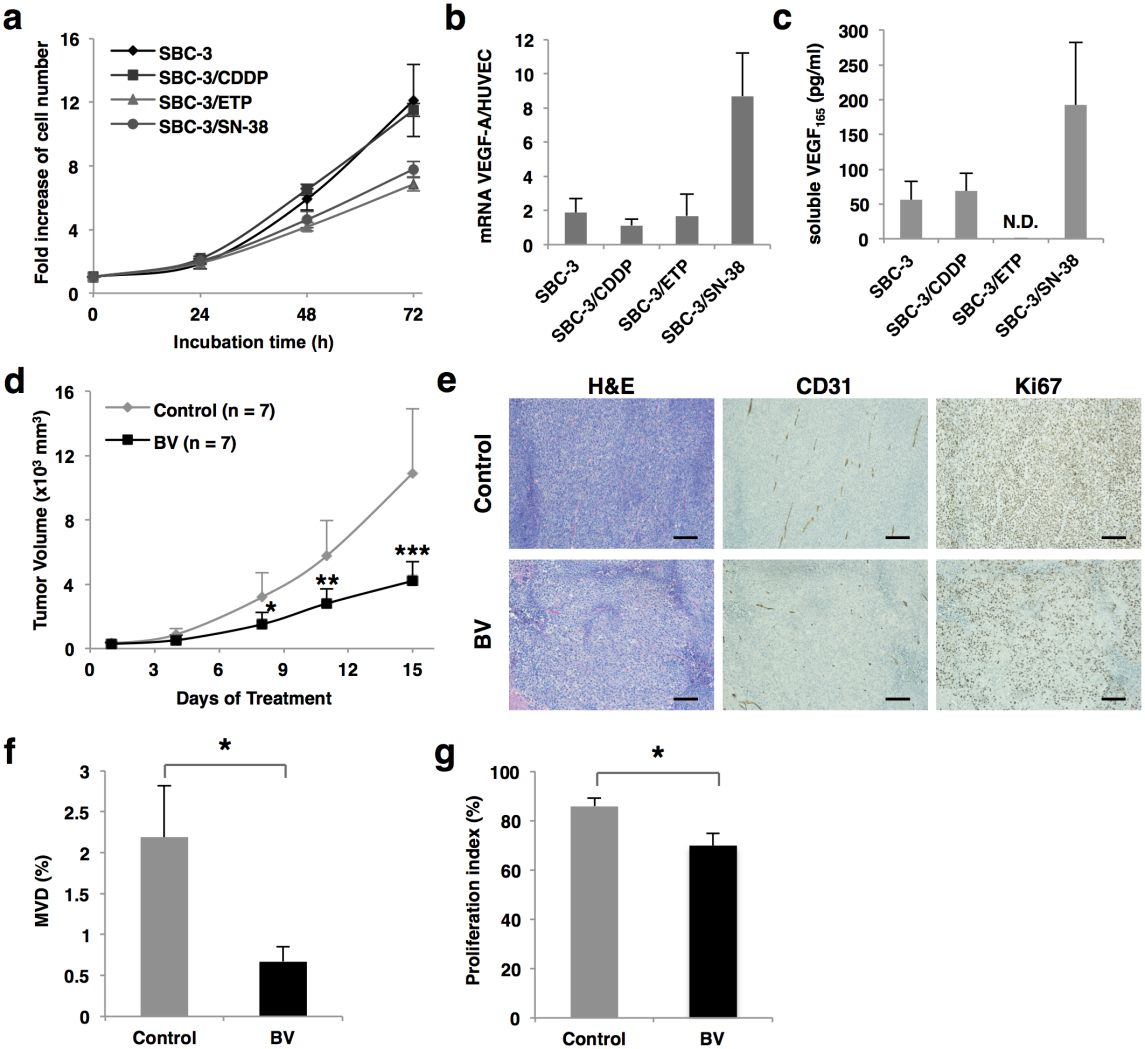
Antitumor effects of bevacizumab against SBC-3/SN-38 xenografts. (a) *In vitro* proliferation assay of parental SBC-3 and SBC-3–derived chemoresistant cells. Points, mean fold increase in cell number; bars, SD from three experiments performed in triplicate. (b) VEGF-A mRNA expression in parental SBC-3 and SBC-3–derived chemoresistant cells was quantified by real-time PCR. Column, mean relative expression ratio to that of HUV-EC-C; bars, SD. (c) Soluble human VEGF_165_ in the supernatants of each SCLC cell line was measured by ELISA. Column, mean concentration; bars, SD. Both experiments were performed at least thrice with triplicate samples. (d) SBC-3/SN-38 cells were inoculated into the flanks of athymic nude mice. When the tumor volume reached approximately 200–300 mm^3^, the mice were randomly assigned to the PBS-treated control arm or bevacizumab (BV)-treated arm (n = 7 mice per group). Bevacizumab was intraperitoneally administered at a dose of 10 mg/kg twice weekly. Points, mean tumor volumes; bars, SD; *, *P* = 0.031; **, *P* = 0.010; ***, *P* = 0.002. (e) Representative H&E and IHC images for CD31 and Ki67. Scale bar, 100 μm. (f) MVD was determined based on the ratio of the CD31-positive area to the total observation area in five randomly selected fields. Column, mean; bars, SD; *, *P* = 0.002. (g) The proliferation index was determined by dividing the number of Ki67-positive cells by the total number of SCLC cells in five randomly selected fields. Column, mean; bars, SD; *, *P* < 0.001.

In order to establish a novel therapeutic strategy for chemoresistant SCLC, another major problem must be solved. Trastuzumab did not show any antitumor effects against SBC-3/SN-38 cells and xenografts (Figure 2d, e and Figure 3a). Although the *in vitro* proliferation rate of SBC-3/SN-38 cells was the same as the parental and other chemoresistant SBC-3 cells (Figure 6a), the *in vivo* growth rate of SBC-3/SN-38 subcutaneous tumors was much greater than other tumors, regardless of trastuzumab treatment (Figure 3a). Lack of ICAM-1 expression may be essential for intrinsic trastuzumab resistance (Figure 4a). Moreover, the rapid growth rate could also explain the resistance. To address this issue, we focused on the discrepancy between the *in vitro* and *in vivo* growth rates. We found that SBC-3/SN-38 cells distinctively produce considerable amounts of VEGF (Figure 6b and c). Since irinotecan is another key drug for SCLC treatment^3^, it is essential to control the growth of SBC-3/SN-38 xenografts for the total treatment of chemoresistant SCLC. Treatment with bevacizumab significantly suppressed the growth of subcutaneous SBC-3/SN-38 tumors in all of seven mice (Figure 6d) with a significant decrease in microvessel density (MVD) and Ki67-positive proliferating cells (Figure 6e–g). On the other hand, bevacizumab treatment could not suppress the growth of SBC-3/ETP xenografts (Supplementary Figure 7), which was likely due to the lack of detectable VEGF production from SBC-3/ETP cells (Figure 6b and c). These results suggest that the antitumor activity of bevacizumab against SBC-3/SN-38 cells depends primarily on the quantity of VEGF produced from tumor cells.

## Discussion

We have shown that the immunomodulatory effects of trastuzumab and antiangiogenic effects of bevacizumab are complementary and can provide a novel therapeutic strategy for HER2-expressing chemoresistant SCLC. The antitumor activity of trastuzumab against HER2-positive MDR SCLC occurs mainly through trastuzumab-mediated ADCC but not through its direct inhibition of HER2 signaling. We also revealed that ICAM-1 and HER2 coexpression on target SCLC cells is indispensable to augment trastuzumab-mediated ADCC. Furthermore, we showed that bevacizumab could salvage trastuzumab-resistance through its antiangiogenic effects.

To date, no molecular targeting therapy has been approved for SCLC, because there are no clinical trials that proved significant clinical benefits^5^,^6^. Although trastuzumab has also been viewed as a promising targeting agent for HER2-positive SCLC^7^,^22^, there has been no preclinical or clinical reports of trastuzumab-based therapy for SCLC. First screening by IHC is useful and crucial for the accurate selection of patients with HER2-targetable SCLC. However, SBC-3 and SBC-3/ETP cells were not or only minimally stained with HercepTest, and were therefore considered negative (Figure 1a and b), although they were HER2-positive by FACS analysis (Supplementary Figure 2b). Moreover, the decision criteria for HER2-positivity with HercepTest differ between gastric and breast cancers^14^,^20^,^23^. These observations imply that many false-negative cases would arise when HercepTest is used to screen SCLC. To address this issue, we established a new IHC technique with higher sensitivity and comparable specificity to HercepTest (Figure 1a and b). Using this IHC method, we identified three HER2-positive cases out of 10 SCLC biopsy specimens.

Regarding the antitumor mechanism of trastuzumab, Clynes et al. clearly demonstrated the importance of ADCC. They showed that much of the antitumor effects of trastuzumab were lost in mice lacking Fcγ receptor^16^. Actually, HER2-positive SCLC cells were lysed by trastuzumab-mediated ADCC (Figure 2d and e), although their proliferation was not inhibited through suppressing HER2 signal transduction (Figure 2a and b). Also *in vivo*, antitumor effects of trastuzumab were considered to be accompanied by tumor-infiltrating immune cells which could induce ADCC (Figure 3a–d).

Notably, the strength of trastuzumab-mediated ADCC activity was not necessarily proportionate to HER2 expression level (Figure 2d and e, Figure 3a, and Supplementary Figure 2b). This phenomenon has also been observed in clinical trials examining the efficacy of trastuzumab for breast cancer. Namely, more than half of patients with HER2 overexpression showed primary resistance^24^,^25^, whereas some patients with normal HER2 expression benefited from trastuzumab^26^. These observations suggest that HER2 expression is not the only predictor of trastuzumab efficacy. Moreover, trastuzumab-mediated ADCC must occur rapidly after HER2 recognition because trastuzumab was internalized and diminished from cell surface shortly after binding to HER2 (Figure 4a–c). Another cell surface molecule is thought to cooperate with HER2 to facilitate and accelerate the completion of trastuzumab-mediated ADCC.

Engagement of LFA-1 by ICAM-1 promotes strong adhesion between effector and target cells^27^, induces polarization of cytolytic granules in NK cells^28^, and costimulates the degranulation of NK cells upon antibody-mediated ligation of CD16 with specific antigen on target cells^29^,^30^. Indeed, SBC-3/ETP (HER2 intermediate/ICAM-1 high) cells were more sensitive than SK-BR-3 (HER2 high/ICAM-1 low) cells to trastuzumab-mediated ADCC (Figure 2d and e, Figure 5a and b, Supplementary Figure 2a and b, and Supplementary Figure 8a and b), and the ADCC activity was canceled by blocking ICAM-1 (Figure 5d). These findings could partly explain why HER2 expression level alone cannot predict the antitumor activity of trastuzumab and imply that ICAM-1 coexpression on target cells is essential for the rapid completion of strong ADCC (Figure 5e).

Since ICAM-1 expression is upregulated when SCLC cells acquire resistance to etoposide (Figure 5a and b, and Supplementary Figure 6), patients with HER2-positive and etoposide-resistant SCLC are ideal candidates for trastuzumab-based therapy. Therefore, future clinical applications of trastuzumab for SCLC should consider combining an initial screening for HER2-positive cases using our highly sensitive IHC method with a secondary screening for ICAM-1–positive cases. Moreover, second-look biopsy is probably more informative to confirm whether HER2 and ICAM-1 expressions are upregulated or homogeneous after acquisition of chemoresistance.

In addition to lack of ICAM-1 expression, upregulation of VEGF production from SCLC cells may be another cause for trastuzumab-resistance. We showed that irinotecan-resistant SCLC cells acquired the potential to produce abundant VEGF (Figure 6b and c). Bevacizumab could salvage irinotecan-resistance by inhibiting VEGF produced from tumor cells (Figure 6d). The efficacy of bevacizumab in combination with cytotoxic drugs in previously untreated SCLC was recently investigated^31^,^32^. In these studies, bevacizumab combination therapy improved response rate and progression-free survival, but failed to improve overall survival. However, based on our findings, bevacizumab could be an attractive agent when administered to patients not in front-line but in relapsed stage especially after irinotecan-containing regimens, where its target VEGF exists abundantly.

**Figure 7:**
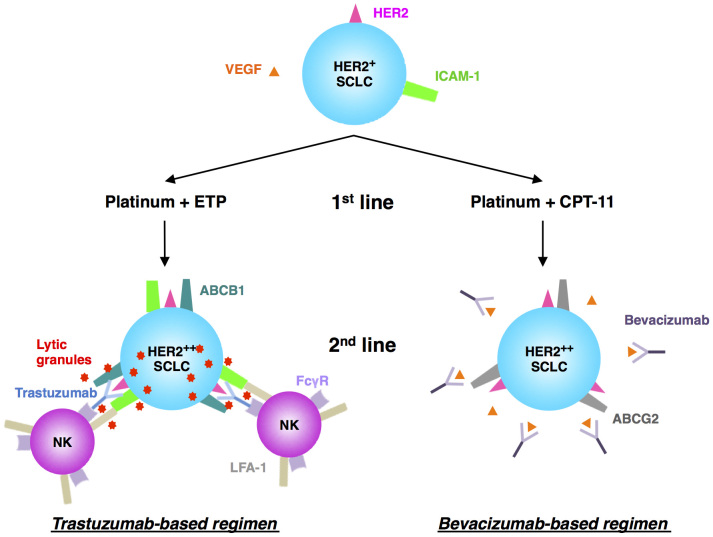
Novel therapeutic decision tree to overcome chemoresistant SCLC based on HER2 expression. After HER2 is detected by IHC at the time of diagnosis, patients are divided into two groups according to the 1^st^ line chemotherapy. Trastuzumab- or bevacizumab-based 2^nd^ line chemotherapy is ideal for patients who have acquired resistance to etoposide-based or irinotecan-based 1^st^ line chemotherapy, respectively. Upregulation of both HER2 and ICAM-1 in etoposide-resistant SCLC cells facilitates trastuzumab-mediated ADCC. Abundant VEGF produced from irinotecan-resistant SCLC cells is the main target of bevacizumab.

Finally, we propose a new stepwise approach for SCLC treatment (Figure 7). This new therapeutic approach focusing on MDR relapsed-SCLC for which clinically beneficial standard therapy has not been established is promising to improve the prognosis of SCLC^4^. Patients with HER2-positive SCLC should be selected prior to front-line chemotherapy. When they become resistant to front-line platinum plus either etoposide or irinotecan, trastuzumab- or bevacizumab-containing regimen should be recommended as the second-line therapy, respectively. If these patients acquire further resistance to all of the pretreated drugs, combination of lapatinib, a dual tyrosine kinase inhibitor of EGFR and HER2, and cytotoxic agents could be considered because lapatinib reverses ATP-binding cassette transporter-mediated chemoresistance in SCLC^19^. Alternative therapy with amrubicin, another optional cytotoxic drug for relapsed SCLC, plus either bevacizumab or trastuzumab may also be beneficial to patients who are naïve to either agent.

Recently, the antibody-cytotoxic drug conjugate T-DM1, composed of trastuzumab and emtansine (DM1), was developed and shown to overcome trastuzumab and lapatinib resistance in heavily pretreated HER2-positive metastatic breast cancers^33^. T-DM1 forms a complex with HER2 and undergoes internalization followed by lysosomal degradation. This process results in an intracellular release of DM1 that exerts cytotoxic activity^34^. Moreover, T-DM1 retains all the mechanisms of action of trastuzumab including ADCC^35^. Trastuzumab is rapidly internalized and undergoes lysosomal degradation in SCLC (Figure 4a–c). Although rapid internalization and lysosomal degradation of T-DM1 is disadvantageous for ADCC induction, this process is beneficial because it exerts strong cytotoxic effects in target cells. In this sense, T-DM1 is expected to show antitumor activity even for patients who did not exhibit trastuzumab-mediated ADCC. Thus, T-DM1 is another attractive therapeutic option for HER2-positive MDR SCLC. Collectively, immunomodulation by trastuzumab and antiangiogenesis by bevacizumab are promising strategies for the treatment of chemoresistant SCLC and might have potential to improve the survival of SCLC patients.

## Methods

### Clinical SCLC tissue samples

SCLC tissue samples were obtained by transbronchial or surgical biopsy from patients at Osaka University Hospital after institutional review board approval. All the patients provided written informed consent.

### Antibodies and reagents

Trastuzumab and bevacizumab were provided by Chugai Pharmaceutical Co. (Tokyo, Japan). Rabbit polyclonal antibodies (Abs) against ubiquitin and actin (Santa Cruz Biotechnology, Santa Cruz, CA), Akt, phospho-Erk, and Erk (Cell Signaling Technology, Danvers, MA), and CD31 and Ki67 (Abcam, Cambridge, England) were used. Mouse monoclonal Abs against CD16 (3G8) and CD54 (HCD54) (BioLegend, San Diego, CA) and CD11a (Ancell, North Bayport, MN), rabbit monoclonal Abs against HER2 (D8F12), phospho-HER2 (6B12) and phospho-Akt (D9E) (Cell Signaling Technology), and rat monoclonal Ab against CD18 (YFC118.3) (Chemicon International, Temecula, CA) are also commercially available. Normal human IgG (Bethyl, Montgomery, TX) and horseradish peroxidase–conjugated goat anti-rabbit or anti-mouse IgG (BioRad, Hercules, CA) were also used. Cell Counting Kit-8 (CCK-8) and Cytotoxicity Detection Kit were obtained from Dojindo (Osaka, Japan) and Roche Applied Science (Penzberg, Upper Bavaria, Germany), respectively.

### Cell lines and cell culture

The biological properties and the origin of SCLC cell lines, including H69, H446, N231, SBC-1, SBC-2, SBC-3, SBC-5, OS-1, OS2RA, OS3R5, Smk, OC-10, and CADO LC6 as well as a breast carcinoma cell line, SK-BR-3, were previously described^19^,^36^. Chemoresistant sublines H69/CDDP^37^ and H69/VP^38^ were obtained from the National Cancer Center (Tokyo, Japan), while SBC-3/CDDP^39^, SBC-3/ETP^40^, and SBC-3/SN-38^41^ were provided by Dr. K. Kiura (Okayama University, Okayama, Japan). A malignant non-Hodgkin's lymphoma cell line, NK92MI, was purchased from American Type Culture Collection (Rockville, MD). The characteristics of the natural killer leukemia cell lines YTS, a subline of YT, and NKL have been previously described^42^,^43^,^44^. HUV-EC-C cells were obtained from the Japan Health Sciences Foundation (Osaka, Japan). All SCLC cells, NKL cells, and YTS cells were maintained in RPMI 1640, and SK-BR-3 cells were maintained in McCOY's 5a supplemented with 10% heat-inactivated fetal bovine serum (FBS), penicillin (100 units/ml), and streptomycin (100 μg/ml). Interleukin-2 (100 units/ml), 1% sodium pyruvate, and 1% non-essential amino acids were also added to the NKL culture medium. HUV-EC-C cells were cultured in EBM-2 medium (Cambrex, Charles City, IA) supplemented with an EGM-2 kit containing 2% FBS, 0.04% hydrocortisone, 0.4% human basic fibroblast growth factor, 0.1% VEGF, and 0.1% heparin.

### Immunohistochemistry (IHC)

Four-μm thick sections were deparaffinized and incubated in Target Retrieval Solution pH 9.0 (Dako, Glostrup, Denmark) for 40 min at 96°C for antigen retrieval, after which endogenous peroxidase activity was blocked with Dako REAL peroxidase blocking (Dako). The sections were allowed to react with diluted primary Abs (1:50–100) overnight. Then, the slides were incubated with a peroxidase-labeled polymer conjugated to secondary anti-rabbit Abs using EnVision™+/HRP (Dako) and developed with 3,3′-deaminobenzidine as the chromogen. HercepTest (Dako) was performed according to the manufacturer's instructions.

### Flow cytometry

To directly detect HER2 using trastuzumab, cells (2 × 10^5^) were incubated with 1 μg of trastuzumab or control human IgG for 45 min at 4°C and then labeled with FITC-conjugated goat anti-human IgG (Abcam). To detect other cell surface proteins (CD54, CD16, CD11a, and CD18), cells (2 × 10^5^) were stained with each properly diluted Ab (1:50–100) for 45 min at 4°C and then labeled with a FITC- or PE-conjugated secondary Ab. Stained cells were analyzed by FACSort (Becton Dickinson, Franklin Lakes, NJ). For CD16 detection, NK cells were preincubated with or without 10 μg/ml of trastuzumab for 4 h.

### Trastuzumab sensitivity and cell proliferation assay

To evaluate the trastuzumab sensitivity of HER2-positive SCLC and SK-BR-3 cells, the cells (5 × 10^3^ cells/well) were plated into 96-well tissue culture–treated plates (Corning) and treated with serially diluted trastuzumab or human IgG in serum-containing medium for 72 h. In the cell proliferation assay, the relative number of viable cells was quantified every 24 h using CCK-8 according to the manufacturer's instructions.

### ADCC assay

In the ADCC assay, NK cell lines, including YTS, NKL^42^,^43^,^44^, and NK92MI cells, were used as effector cells (E). In particular, CD16-positive YTS cells were sorted using FACSAria (Becton Dickinson) prior to the assay. SCLC cells (5 × 10^3^ cells/well) as the target cells (T) were coincubated with NK cells at various E/T ratios with 10 μg/ml of trastuzumab or normal IgG in triplicate in 96-well plates. In the ICAM-1–blocking assay, 10 μg/ml of an anti-CD54–blocking Ab was also added. After 4-h incubation at 37°C, lactate dehydrogenase activity in the cell-free supernatants was measured using a Cytotoxicity Detection Kit and cytotoxicity was calculated according to manufacturer's instructions.

### Trastuzumab internalization analysis

Cell surface–binding trastuzumab was analyzed by FACS as previously described^45^. Briefly, SCLC cells were incubated with 10 μg/ml of trastuzumab for 45 min at 4°C, and then either warmed to 37°C to allow internalization or maintained at 4°C. After internalization was stopped by transferring the cells to ice-cold buffer, cells were stained with FITC-conjugated goat anti-human IgG (Abcam) and analyzed by FACSort. The internalization rate was calculated as the mean fluorescence intensity (MFI) of the trastuzumab occupancy relative to that at the beginning of the internalization period using the formula [(MFI _measured_ _trastuzumab occupancy_ − background)/(MFI _0 minutes_ _trastuzumab occupancy_ − background)] × 100%.

To visualize trastuzumab internalization, cells grown on polyethyleneimine-coated cover glass (ASAHI GLASS, Tokyo, Japan) in 24-well plates (Corning, Corning, NY) were incubated with 50 nmol/l of Lysotracker (Lonza, Walkersville, MD) for 60 min at 37°C and then stained with 10 μg/ml of Alexa 488-labeled trastuzumab using an Alexa Fluor 488 monoclonal Ab labeling kit (Molecular Probes, Eugene, OR). Digital video images were collected under BZ-9000 (KEYENCE, Osaka, Japan) for up to 120 min and then analyzed using BZ-H2A software.

### Immunoprecipitation and immunoblotting

Cells were either left untreated or treated with 10 μg/ml of trastuzumab for up to 240 min, and then lysed in lysis buffer. For immunoprecipitation analyses, whole cell lysates were precleared with 20 μl of protein G sepharose bead slurry (GE Healthcare, Buckinghamshire, UK), and then incubated with an anti-ubiquitin Ab (diluted 1:40) overnight at 4°C with gentle agitation. The immunoprecipitates were washed thrice in lysis buffer, denatured, and separated on a 5–20% gradient gel (Wako, Osaka, Japan) by SDS-PAGE and then transferred to a polyvinylidene difluoride membrane. The proteins were immunoblotted with an anti-HER2 rabbit monoclonal Ab (diluted 1:500) overnight at 4°C followed by the appropriate horseradish peroxidase–conjugated secondary Abs (diluted 1:10,000) for 1 h at room temperature. Immunoreactive bands were visualized using a chemiluminescent technique with ECL Plus Western Blotting Detection Reagents (GE Healthcare).

### Quantitative reverse-transcription polymerase chain reaction (qRT-PCR)

Total RNA was extracted using an RNeasy Mini Kit (Qiagen, Valencia, CA), and cDNA was synthesized from 1 μg total RNA using High Capacity cDNA Reverse Transcription Kits (Applied Biosystems, Carlsbad, CA). qRT-PCR was performed with an Applied Biosystems Prism 7900HT Sequence Detection System using TaqMan® Universal PCR master mix according to the manufacturer's specifications (Applied Biosystems). The assay ID for the TaqMan primer set for human *VEGF-A* was no. Hs99999090_m1 (Applied Biosystems). Human *GAPDH* was used as an endogenous control (Applied Biosystems, Part Number 4352934E). Amplifications were performed in duplicate in ABI7900HT 96-well microtiter plates (Applied Biosystems). The thermal cycling conditions for the ABI7900HT were as follows: AmpErase UNG Activation for 2 min at 50°C, AmpliTaq God DNA Polymerase Activation for 10 min at 95°C, followed by 40 cycles each of Melt Anneal/Extend for 15 sec at 95°C and 1 min at 60°C.

### Enzyme-linked immunosorbent assay (ELISA)

Cells (3 × 10^4^ cells/well) were plated into 24-well tissue culture–treated plates (Corning) in triplicate and allowed to proliferate for 24 h. Then, the supernatants of each well were collected, and the levels of human VEGF_165_ were measured using Quantikine kits (R&D Systems, Minneapolis, MN) according to the manufacturer's protocol.

### Treatment of HER2-positive SCLC xenografts

All animal experiments were approved by The Institute of Experimental Animal Sciences Osaka University Medical School. Six-to-eight week old male BALB/cA Jcl nu/nu mice were obtained from CLEA Japan (Osaka, Japan). SCLC cells (1–2 × 10^7^) were inoculated subcutaneously into the flanks of mice. When the tumor volume reached approximately 200–300 mm^3^, the mice were randomly assigned into one of two treatment groups and either treated with intraperitoneal injections of PBS or 30 mg/kg of trastuzumab twice weekly to evaluate the antitumor activity of trastuzumab^46^, or PBS or 10 mg/kg of bevacizumab twice weekly to the evaluate antitumor activity of bevacizumab^47^. Tumor volume (V) was calculated using the following equation: V (mm^3^) = length × (width)^2^/2. Mice were sacrificed by CO_2_ inhalation, and tumor samples were resected by the 50^th^ treatment day for histological analyses.

### TUNEL staining

TUNEL staining was performed to measure apoptosis in paraffin-embedded tumor sections using ApopTag Peroxidase In Situ Apoptosis Detection kit according to the manufacturer's instructions (Merck Millipore, Billerica, MA). Cell nuclei were stained with methyl green.

### Micro vessel density (MVD) assessment

MVD (%) was calculated based on the ratio of the CD31-positive area to the total observation area in the viable region. At least five fields per section were randomly analyzed, excluding necrotic areas. Quantification was performed using imaging analysis software (NIS-Elements D3.00, Nicon Instruments Company, Tokyo, Japan).

### Statistical analysis

All statistical evaluations were performed in triplicate for each experiment and repeated at least thrice. Mean ± SD values were calculated, and differences were evaluated using two-sided Student's *t* test. *P* < 0.05 was considered statistically significant.

## Author Contributions

T.M. and T.K. conceived the study and designed experiments; T.M., S.K., Y.O., I.N. and S.I. carried out experiments; T.M., T.K., R.T., K.M., M.H., O.M., K.T. and H.H. collected tumor samples from patients; H.A. and A.K. interpreted and advised on immunological experiments; T.M., T.K., S.K., K.I., Y.T., H.K., I.T. analyzed the data; T.M., T.K. and A.K. wrote the manuscript, which was reviewed and edited by the other coauthors.

## Supplementary Material

Supplementary Information Supplementary Information
