# Supplementary material for: Overcoming chemoresistance of small-cell lung cancer through stepwise HER2-targeted antibody-dependent cell-mediated cytotoxicity and VEGF-targeted antiangiogenesis
Source: Sci Rep. 2013 Sep 16;3:2669. doi: 10.1038/srep02669 (PMC3773623; doi:10.1038/srep02669)

Supplementary Information

Overcoming chemoresistance of small-cell lung cancer through stepwise HER2-targeted antibody-dependent cell-mediated cytotoxicity and VEGF-targeted antiangiogenesis

Toshiyuki Minami[1], Takashi Kijima[*1], Satoshi Kohmo[1], Hisashi Arase[2, 3], Yasushi Otani[1], Izumi Nagatomo[1], Ryo Takahashi[1], Kotaro Miyake[1], Masayoshi Higashiguchi[1], Osamu Morimura[1], Shoichi Ihara[1], Kazuyuki Tsujino[1], Haruhiko Hirata[1], Koji Inoue[1], Yoshito Takeda[1], Hiroshi Kida[1], Isao Tachibana[1], and Atsushi Kumanogoh[1, 3, 4]

Authors' Affiliations:

[1] Department of Respiratory Medicine, Allergy and Rheumatic Diseases, Osaka University Graduate School of Medicine, Osaka, Japan.

[2] Laboratory of Immunochemistry, World Premier International Research Center (WPI), Immunology Frontier Research Center, and Department of Immunochemistry, Research Institute for Microbial Disease, Osaka University, Suita, Osaka, Japan.

[3] Core Research for Evolutional Science and Technology, Japan Science and Technology Agency, 4-1-8, Honcho Kawaguchi, Saitama 332-0012, Japan

[4] Department of Immunopathology, Immunology Frontier Research Center, Osaka University, Osaka, Japan

Supplementary Figure Legends

Supplementary Figure 1. Detection of HER2 expression in human SCLC specimens by D8F12 Ab-based IHC. Three of 10 human SCLC specimens were determined to be HER2-positive (a) while seven were negative (b). Scale bars, 25 µm.

Supplementary Figure 2. HER2 is preferentially expressed in Japanese SCLC and upregulated in SBC-3–derived chemoresistant cells. (a) HER2 expression in 13 SCLC cell lines (H69, H446, and N231 cells are of Caucasian origin, while SBC-1, SBC-2, SBC-3, SBC-5, OS-1, OS2RA, OS3R5, Smk, OC-10, and CADO LC6 cells are of Japanese origin). (b) Comparison of HER2 expression between parental SBC-3 and chemoresistant SBC-3 sublines. In both (a) and (b), FACS histograms are shown for cells stained with 10 µg/ml of either trastuzumab (black shaded) or a control normal human IgG (solid line). Experiments were performed at least twice with similar results.

Supplementary Figure 3. Antitumor effects of trastuzumab on SBC-3/ETP xenografts. SBC-3/ETP cells were inoculated into the flanks of athymic nude mice. When the tumor volume reached approximately 200–300 mm^3, the mice

were randomly assigned to one of two groups, and treated twice weekly with intraperitoneal injections of either PBS (control), 10 mg/kg or 30 mg/kg of trastuzumab (n = 7 per group). *, $P < 0.001$; N.S., not significant.

Supplementary Figure 4. Histological analysis of SBC-3 xenografts. (a) Representative H&E and IHC images for HER2, TUNEL, and CD11b are shown as described in Figure 3b. Scale bar, 50 µm. (b) and (c) Quantification of TUNEL-positive apoptotic cells and CD11b-positive cells. Data are shown as described in Figure 3c and d. N.S., not significant.

Supplementary Figure 5. Expression of LFA-1, an integrin heterodimer consisting of CD11a (α chain) and CD18 (β chain), in NK cell lines (NKL, YTS, and NK92MI cells). FACS histograms are shown for cells that were stained with 5 µg/ml of an anti-human CD11a or CD18 Ab (black shaded) or an isotype-matched control Ab (solid line). Results are representative of at least two experiments.

Supplementary Figure 6. Expression of cell surface ICAM-1 (CD54). ICAM-1 expression in HER2-negative H69 and H69-derived chemoresistant cells (H69/CDDP and H69/VP cells). FACS histograms are shown for cells stained

with 5 µg/ml of an anti-human CD54 Ab (black shaded) or an isotype-matched control Ab (solid line). Results are representative of at least two experiments.

Supplementary Figure 7. Antitumor effects of bevacizumab on SBC-3/ETP xenografts. SBC-3/ETP cells were inoculated into the flanks of athymic nude mice. When the tumor volume reached approximately 200–300 mm^3, the mice were randomly assigned to one of two groups, and treated twice weekly with intraperitoneal injections of either PBS (control) or 10 mg/kg of bevacizumab (BV) (n = 8 per group). N.S., not significant.

Supplementary Figure 8. Expression of cell surface ICAM-1 (CD54) on SK-BR-3 cells. (a) Cell surface ICAM-1 expression in SK-BR-3 cells. FACS results are representative of at least two experiments and are presented as histograms of CD54-stained cells (black shaded) and control cells (solid line). (b) IHC analysis revealed that very few SK-BR-3 cells express ICAM-1. Scale bar, 50 µm.

Supplementary Figure 1

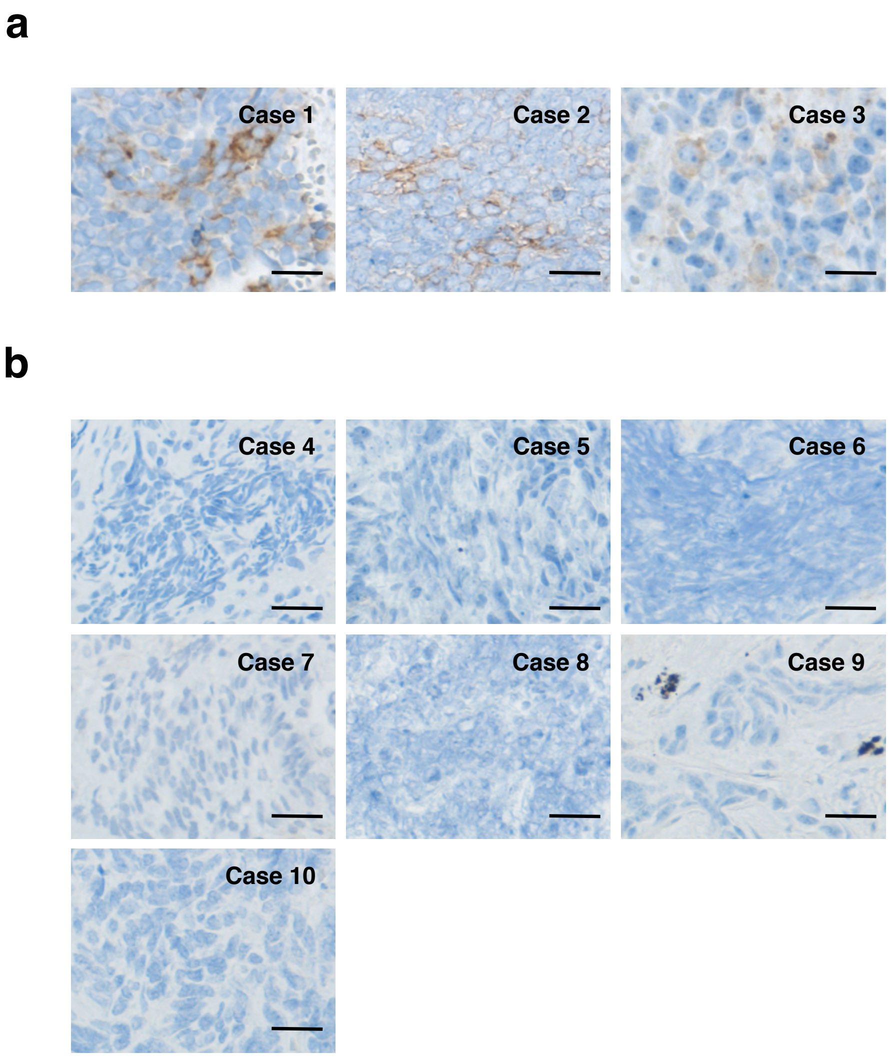

Supplementary Figure 2

a

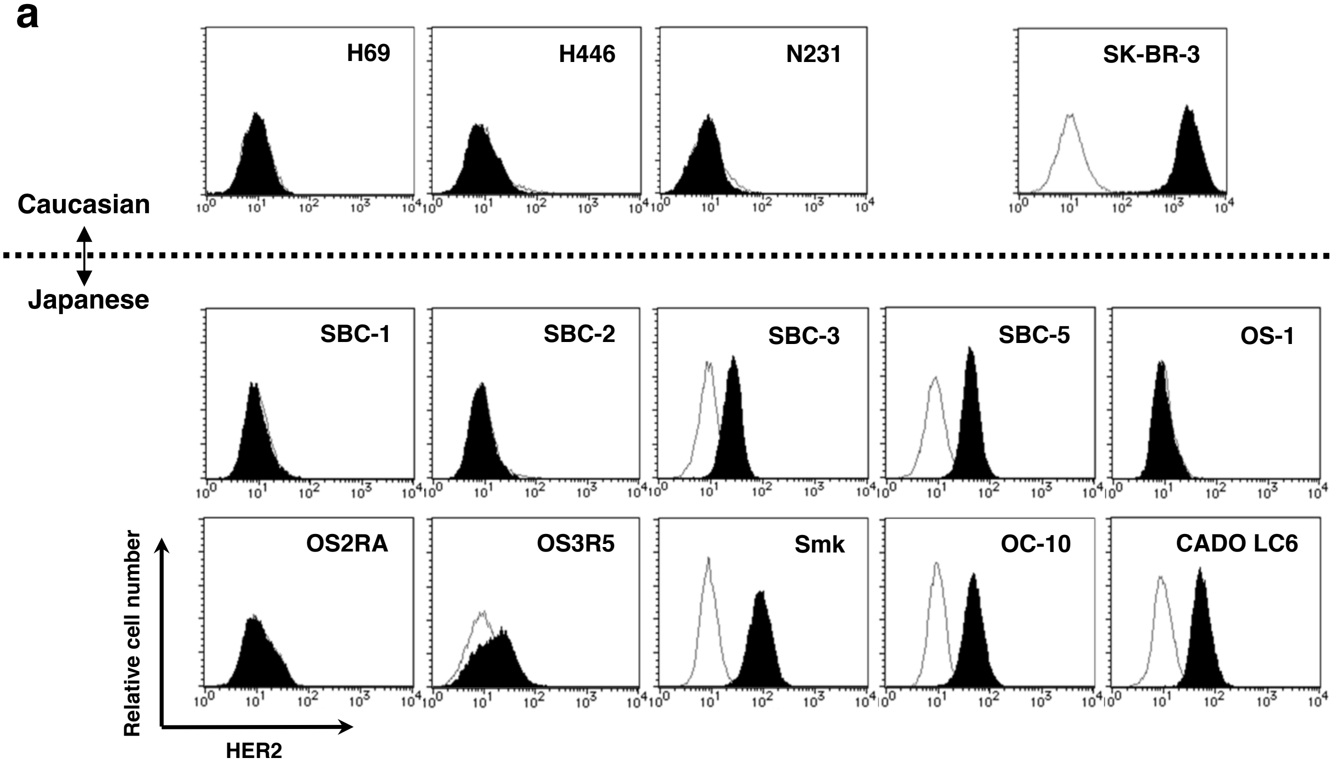

b

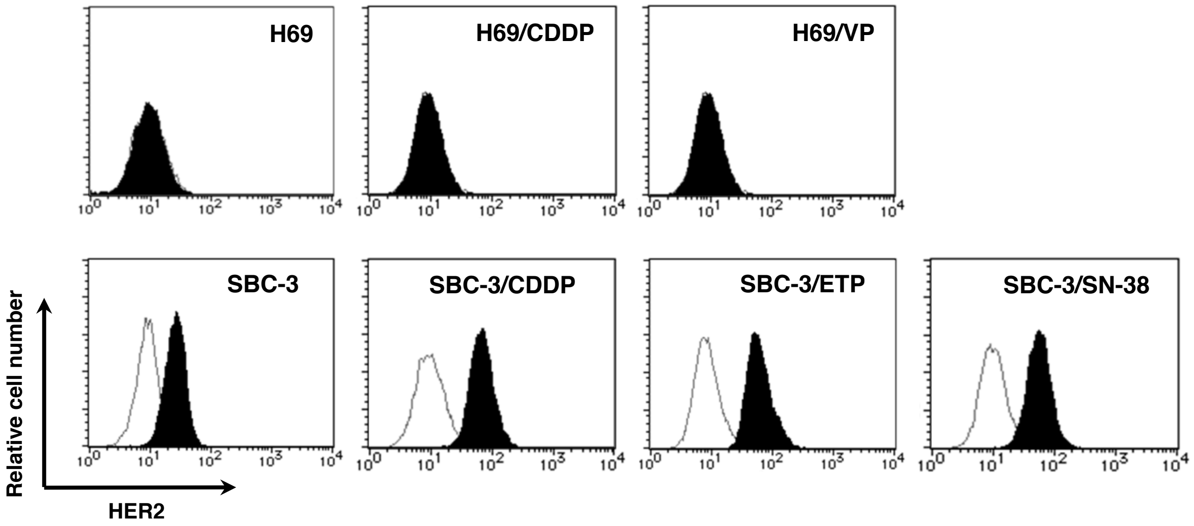

Supplementary Figure 3

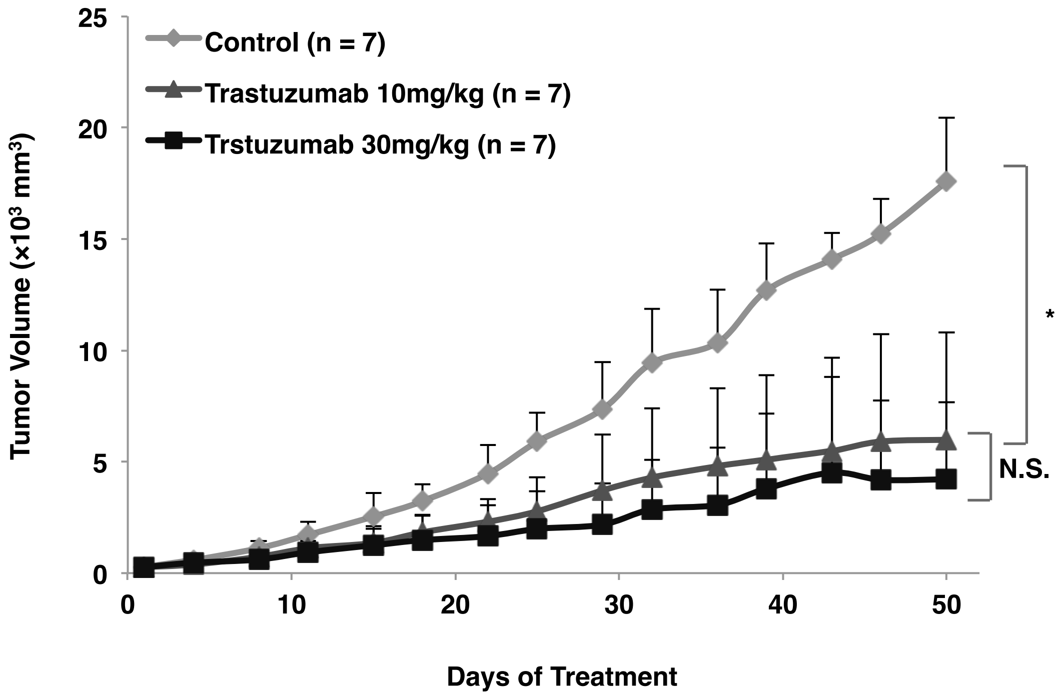

Supplementary Figure 4

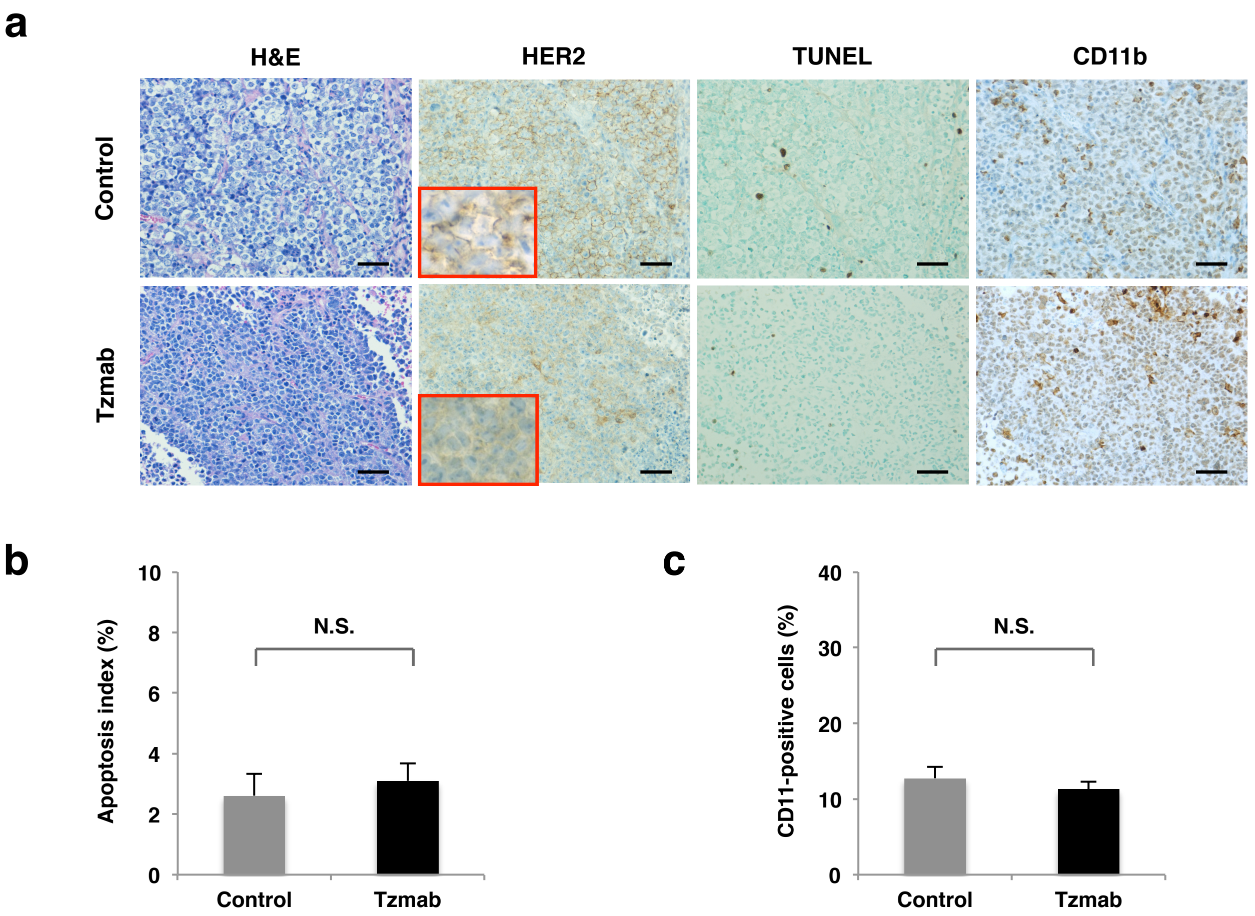

Supplementary Figure 5

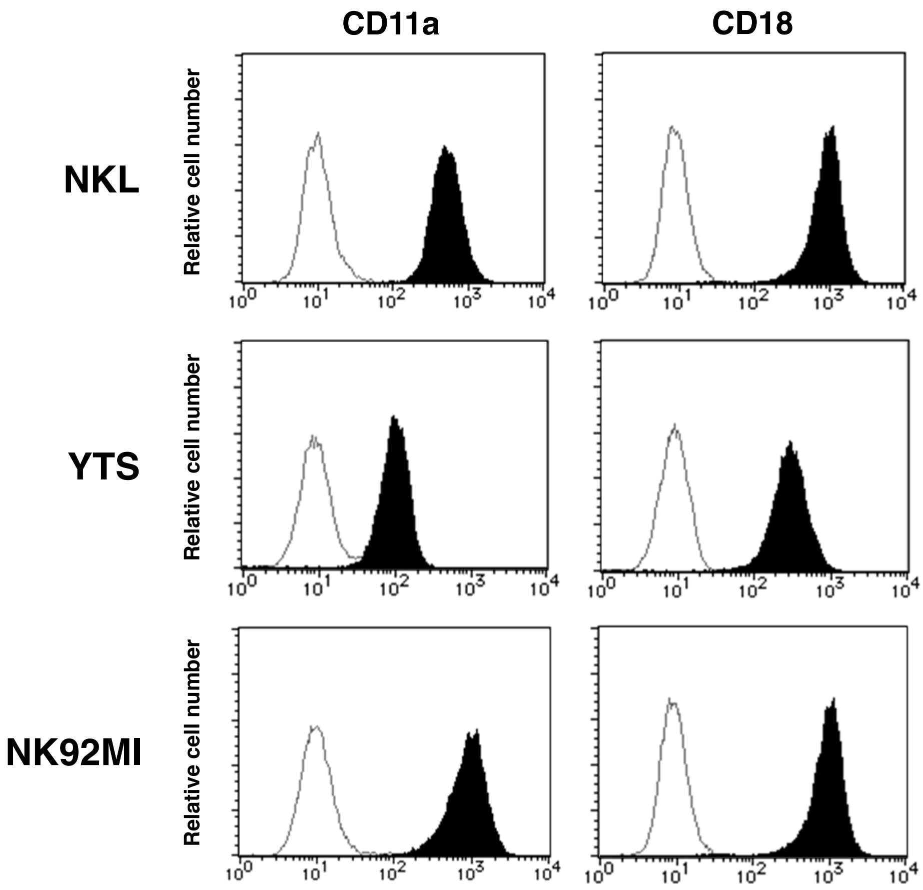

Supplementary Figure 6

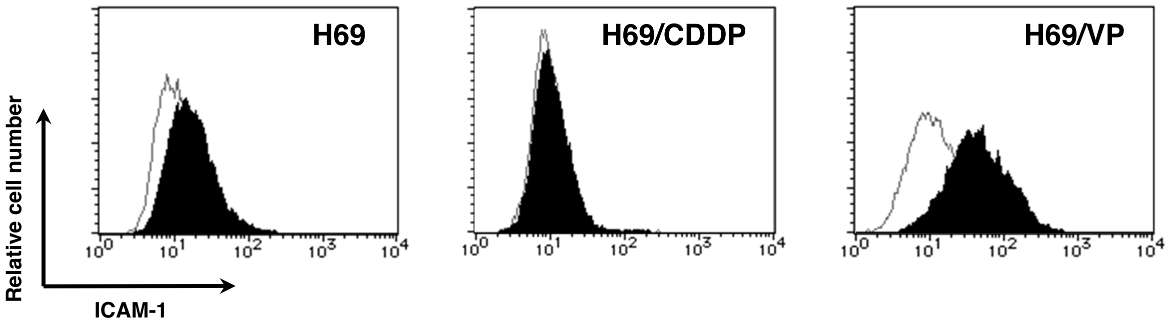

Supplementary Figure 7

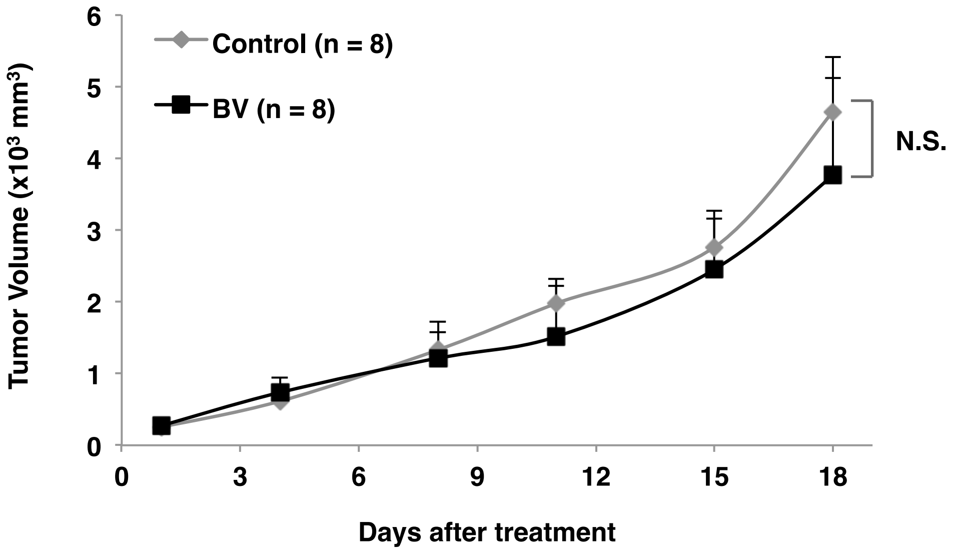

Supplementary Figure 8

a

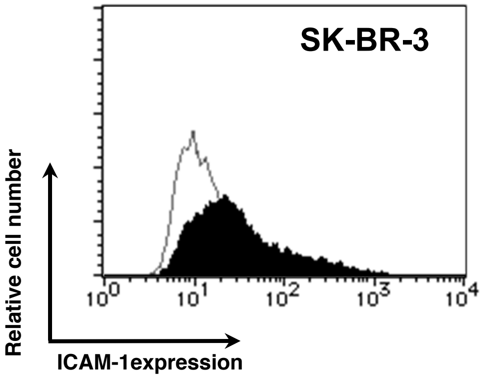

b

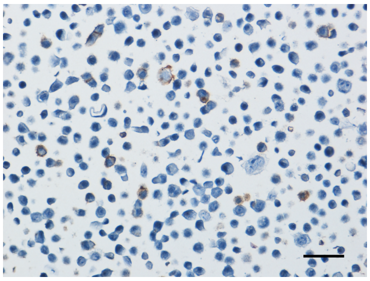

Full-length blot for Figure 2b

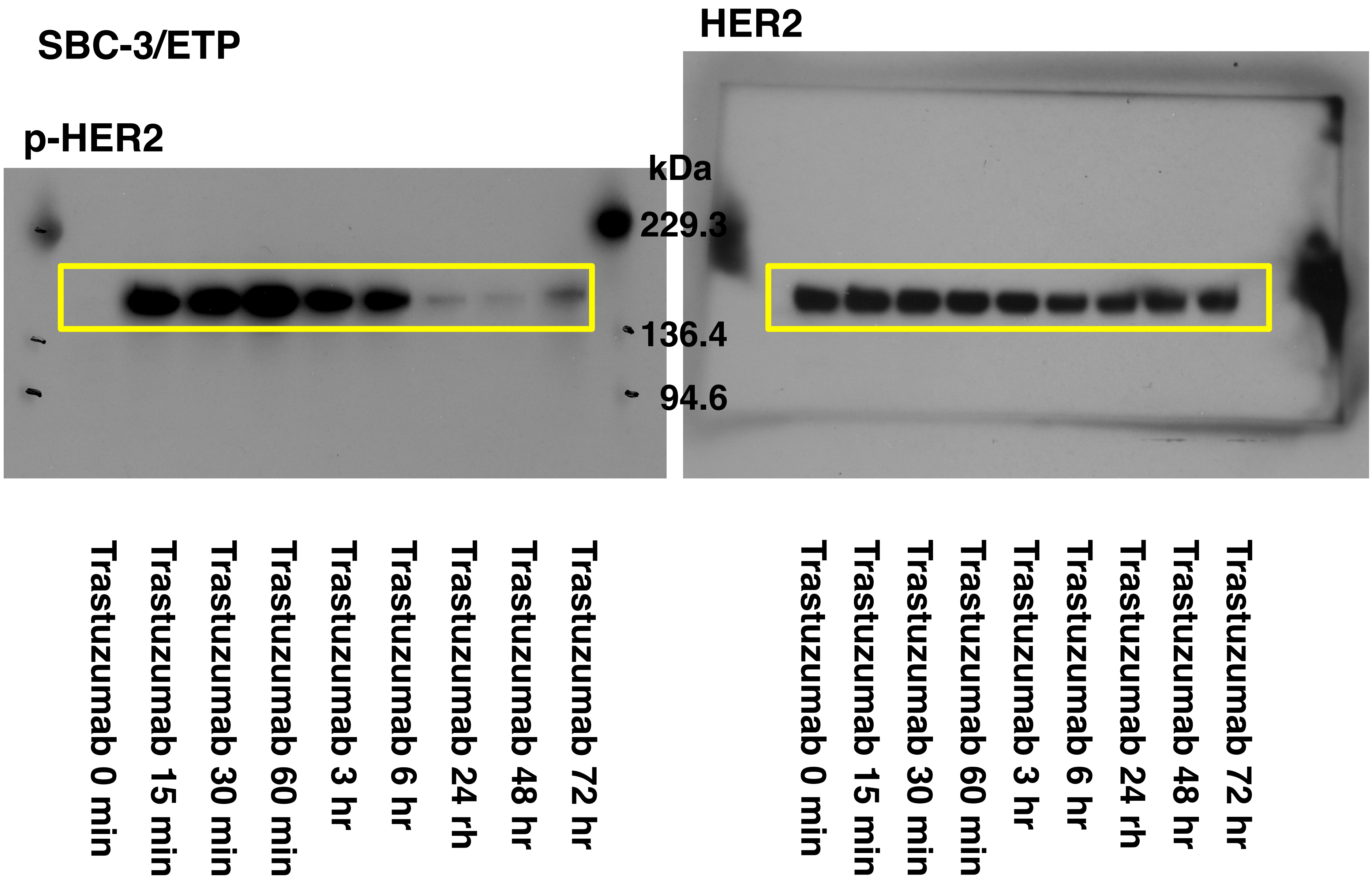

Full-length blot for Figure 2b

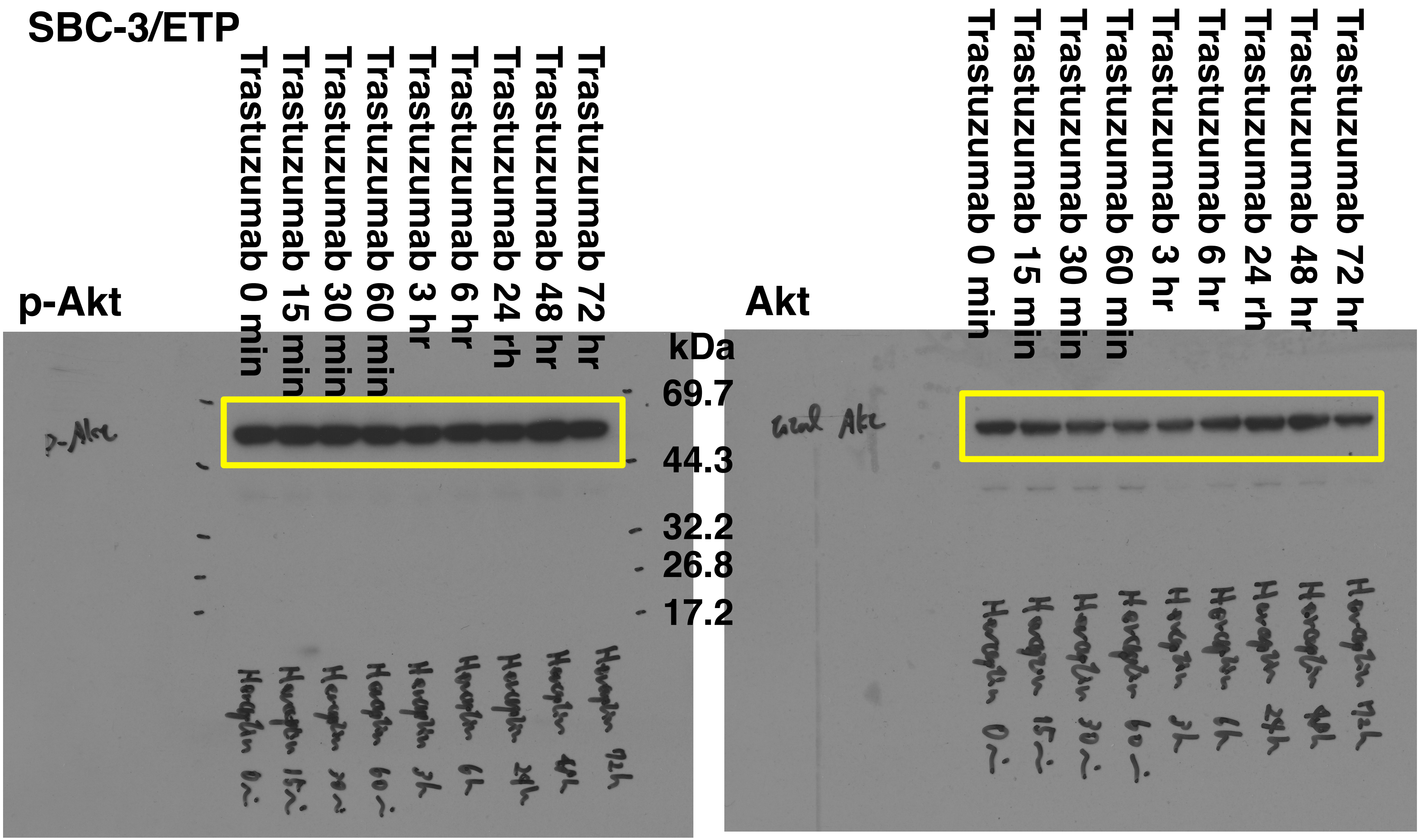

The handwritten word “Herceptin” is a trade name of trastuzumab.

Full-length blot for Figure 2b

SBC-3/ETP

p-Erk

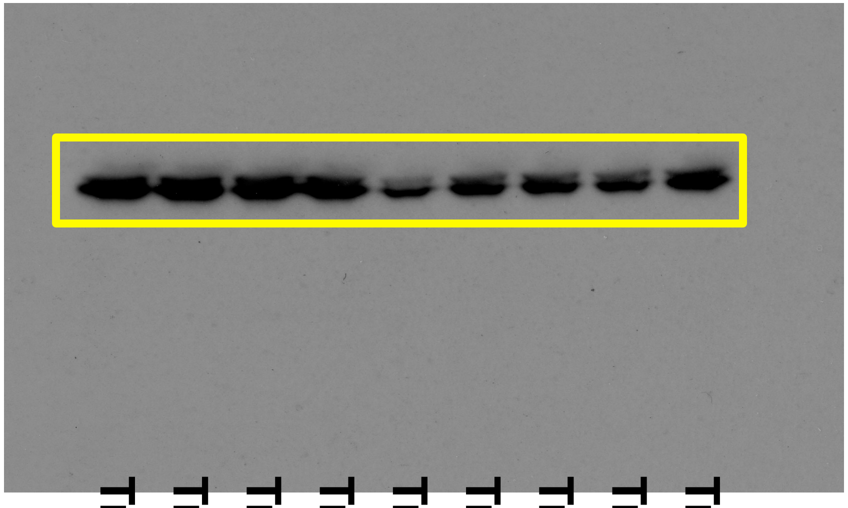

Trastuzumab 0 min
Trastuzumab 15 min
Trastuzumab 30 min
Trastuzumab 60 min
Trastuzumab 3 hr
Trastuzumab 6 hr
Trastuzumab 24 rh
Trastuzumab 48 hr
Trastuzumab 72 hr

Erk

kDa

Trastuzumab 0 min
Trastuzumab 15 min
Trastuzumab 30 min
Trastuzumab 60 min
Trastuzumab 3 hr
Trastuzumab 6 hr
Trastuzumab 24 rh
Trastuzumab 48 hr
Trastuzumab 72 hr

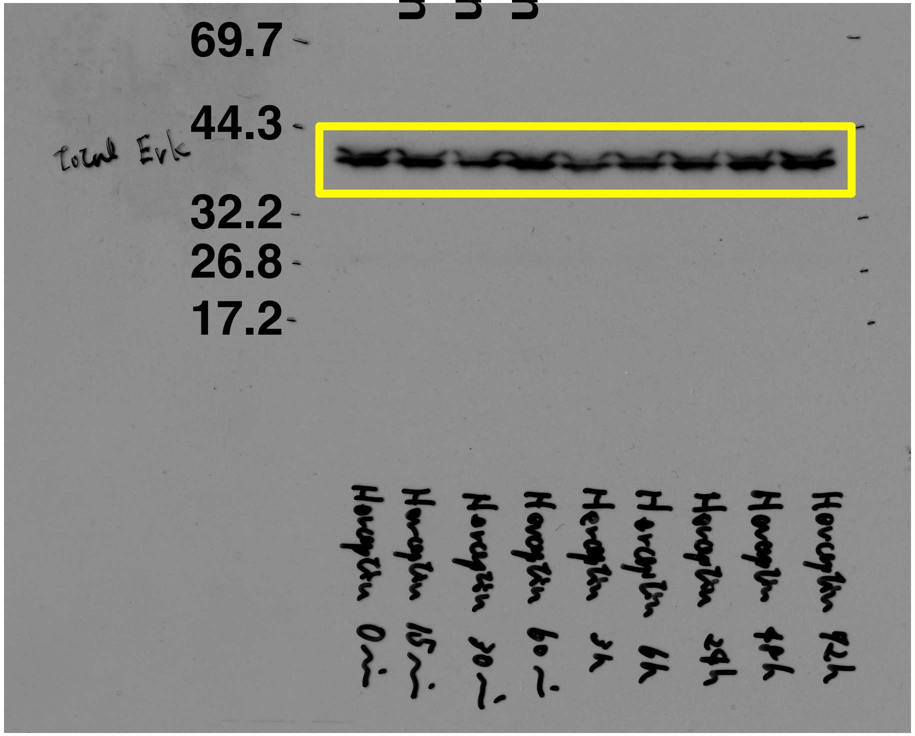

Full-length blot for Figure 2b

SBC-3/ETP

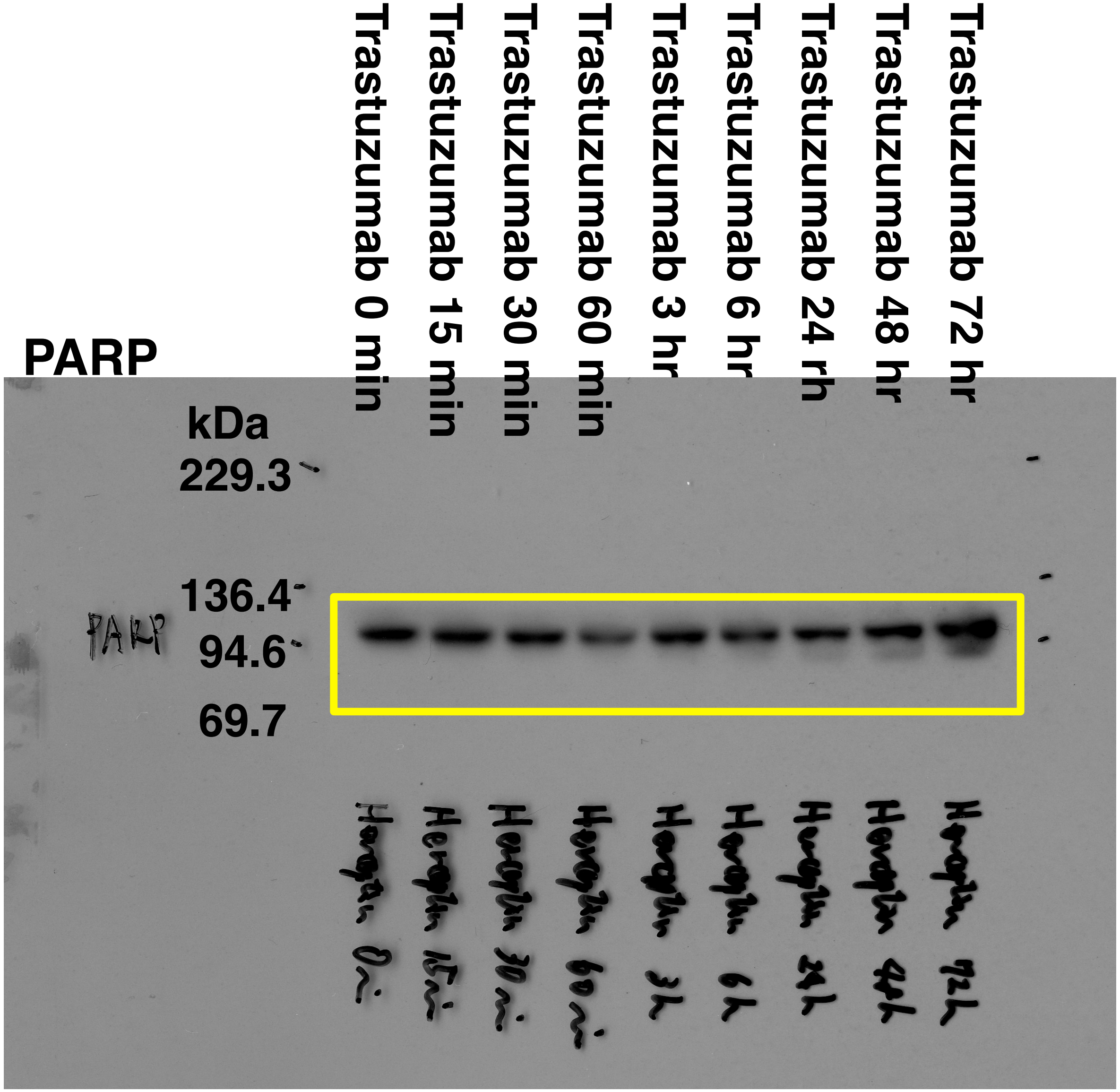

Full-length blot for Figure 2b

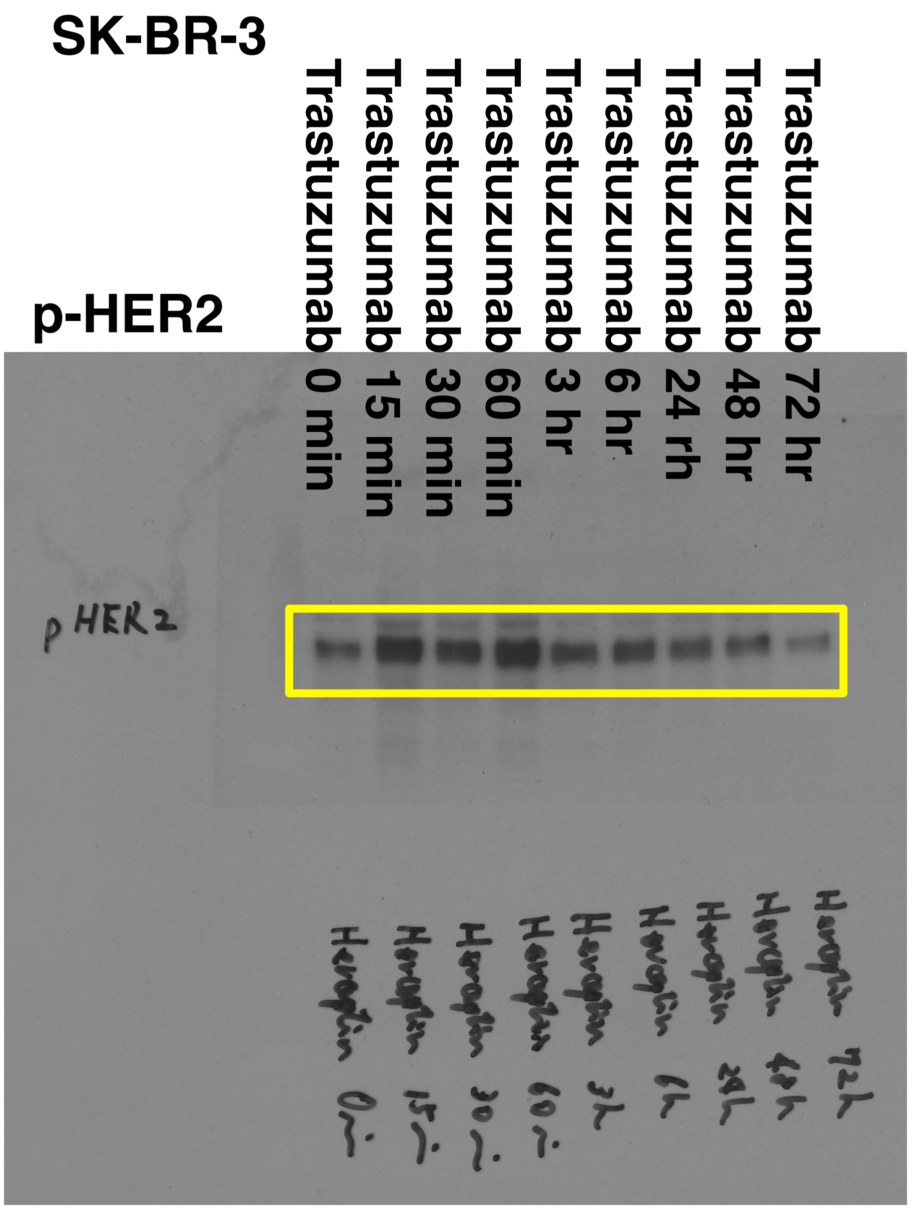

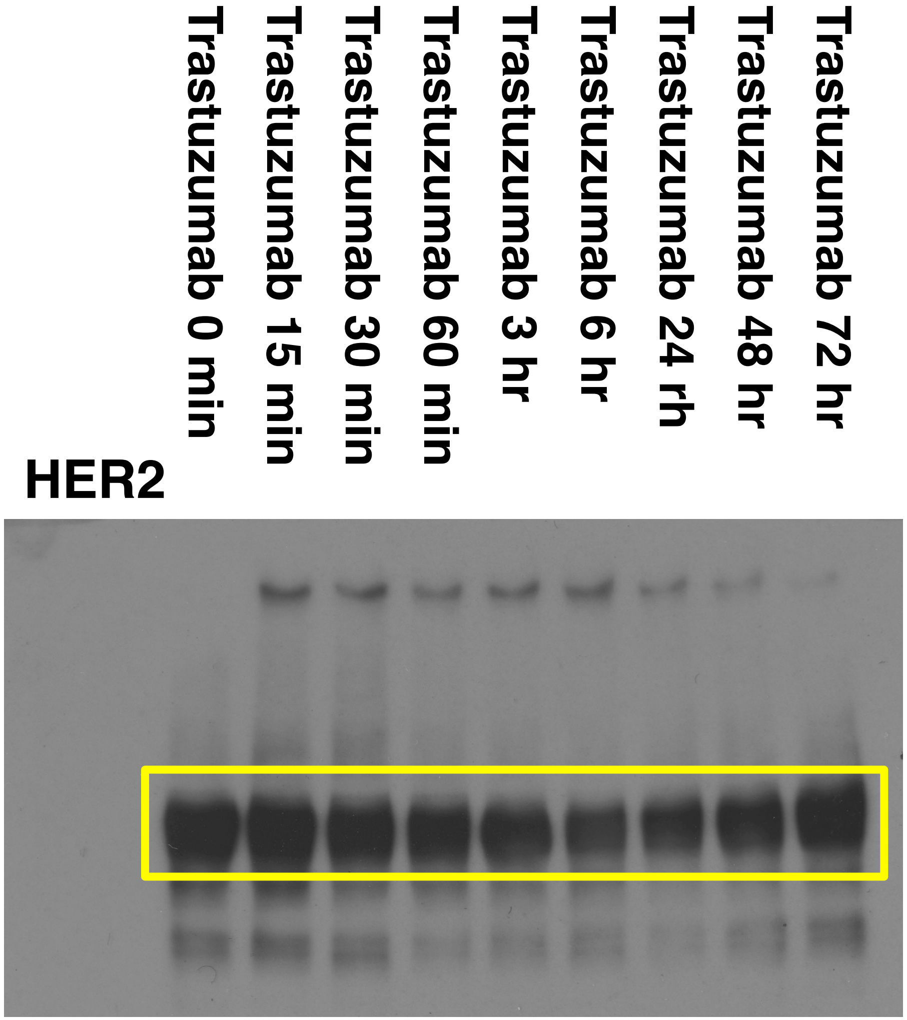

Full-length blot for Figure 2b

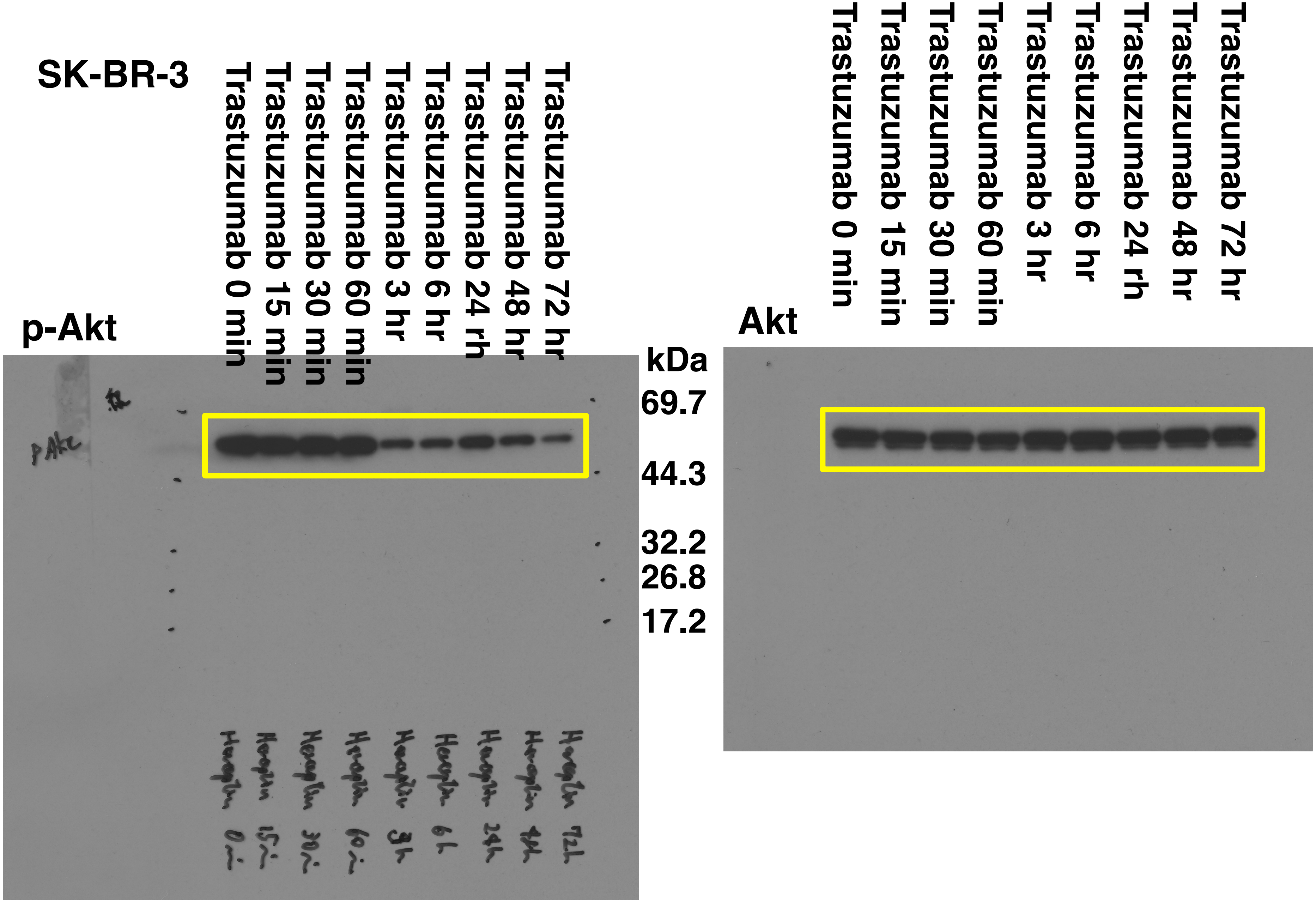

Full-length blot for Figure 2b

SK-BR-3

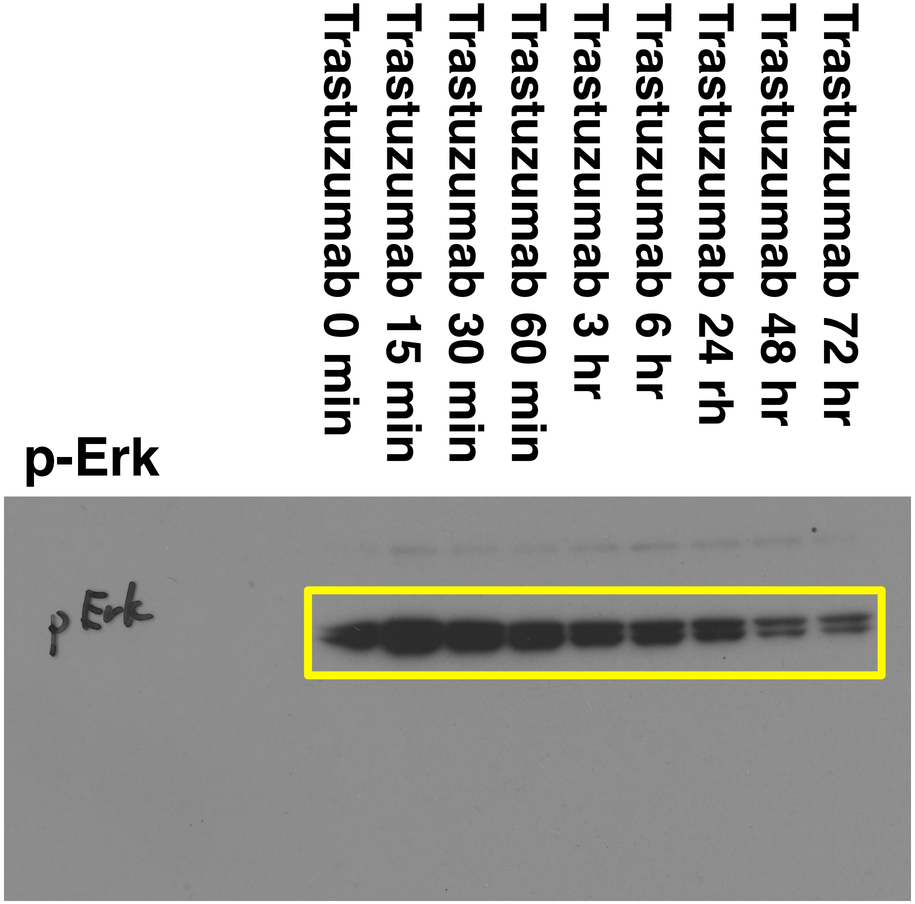

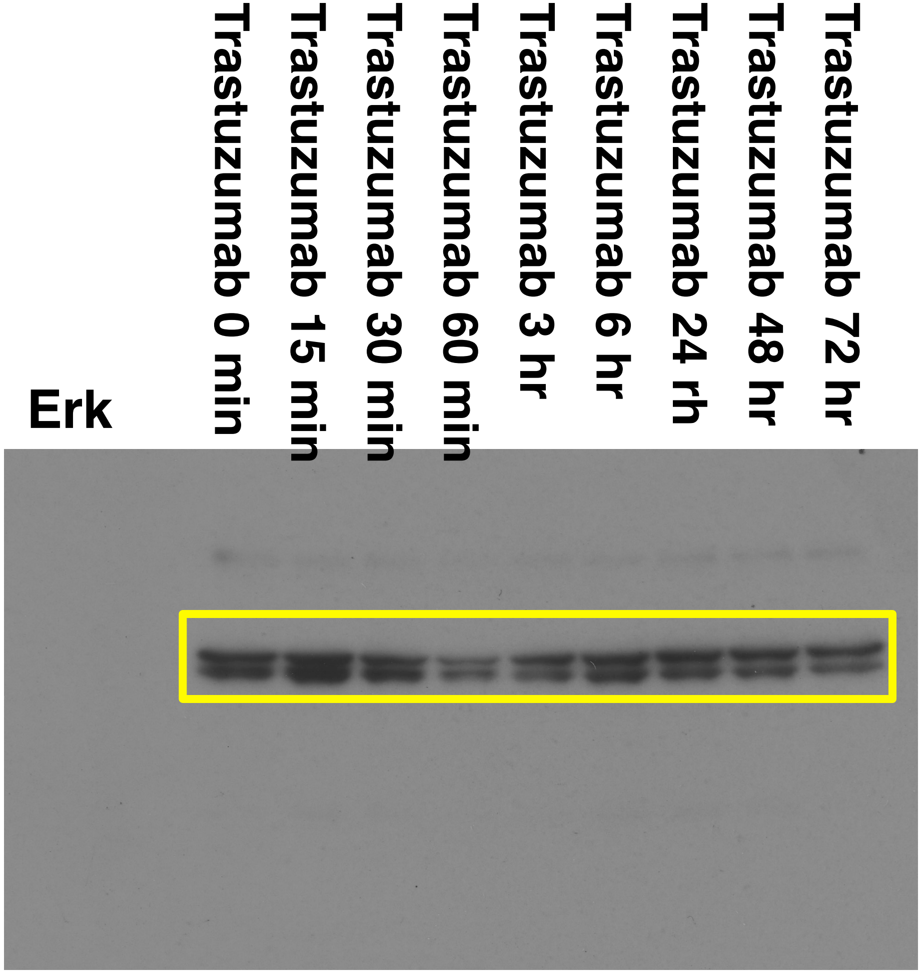

Full-length blot for Figure 2b

SK-BR-3

PARP

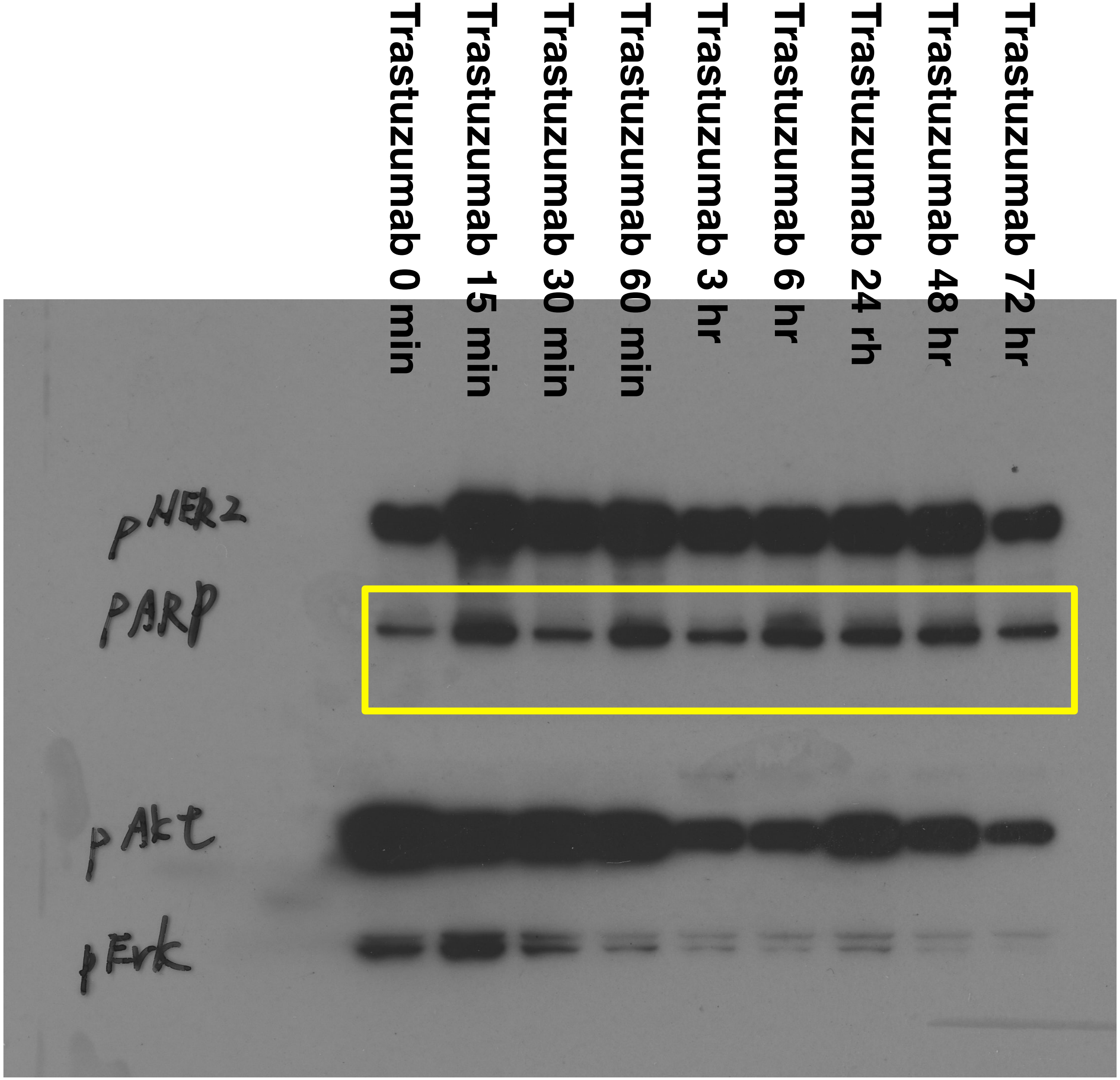

Full-length blot for Figure 4b

SBC-3 IP: ubiquitin IB: HER2 **SBC-3/CDDP IP: ubiquitin IB: HER2**

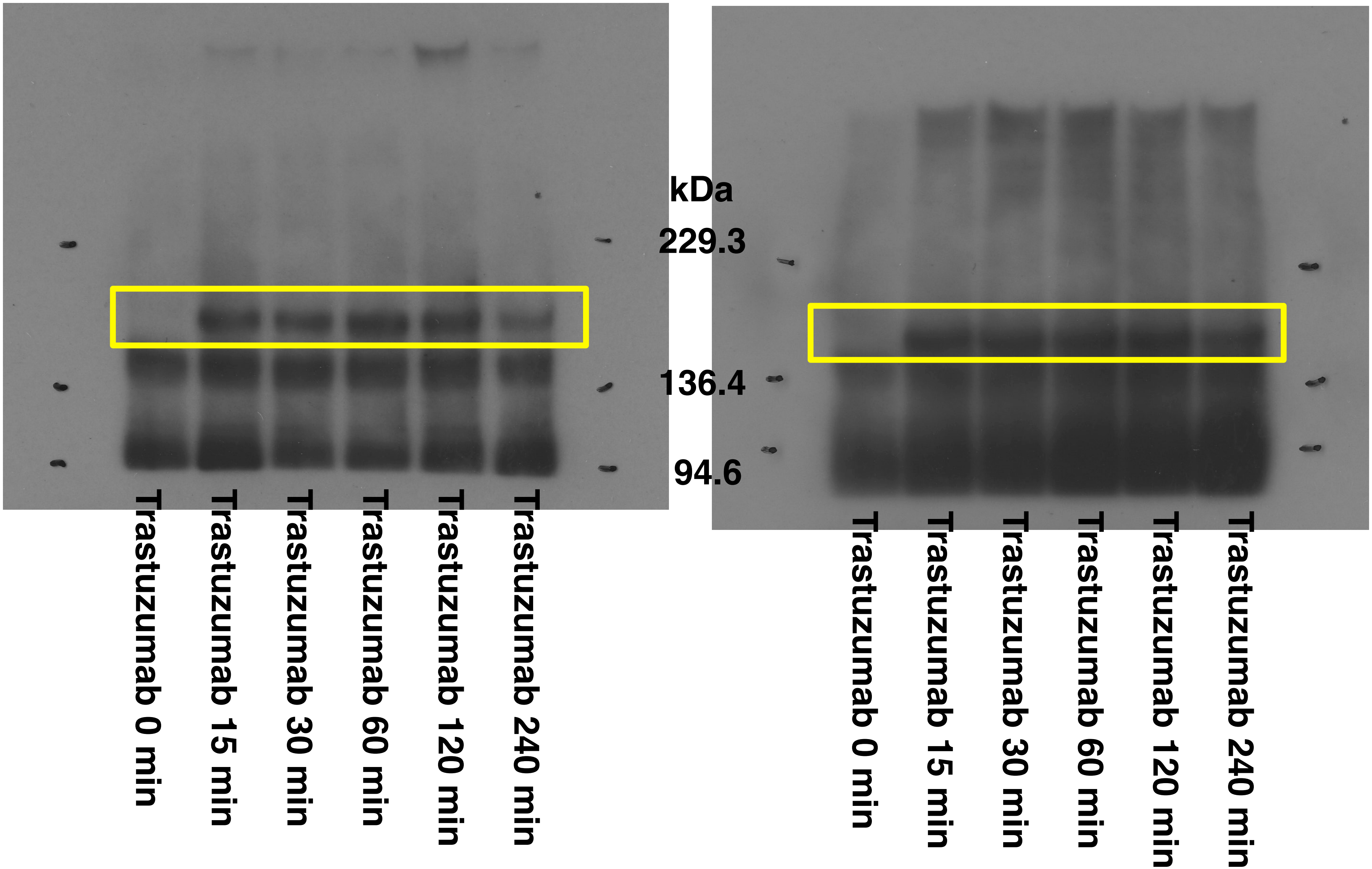

Full-length blot for Figure 4b

SBC-3/ETP IP: ubiquitin IB: HER2

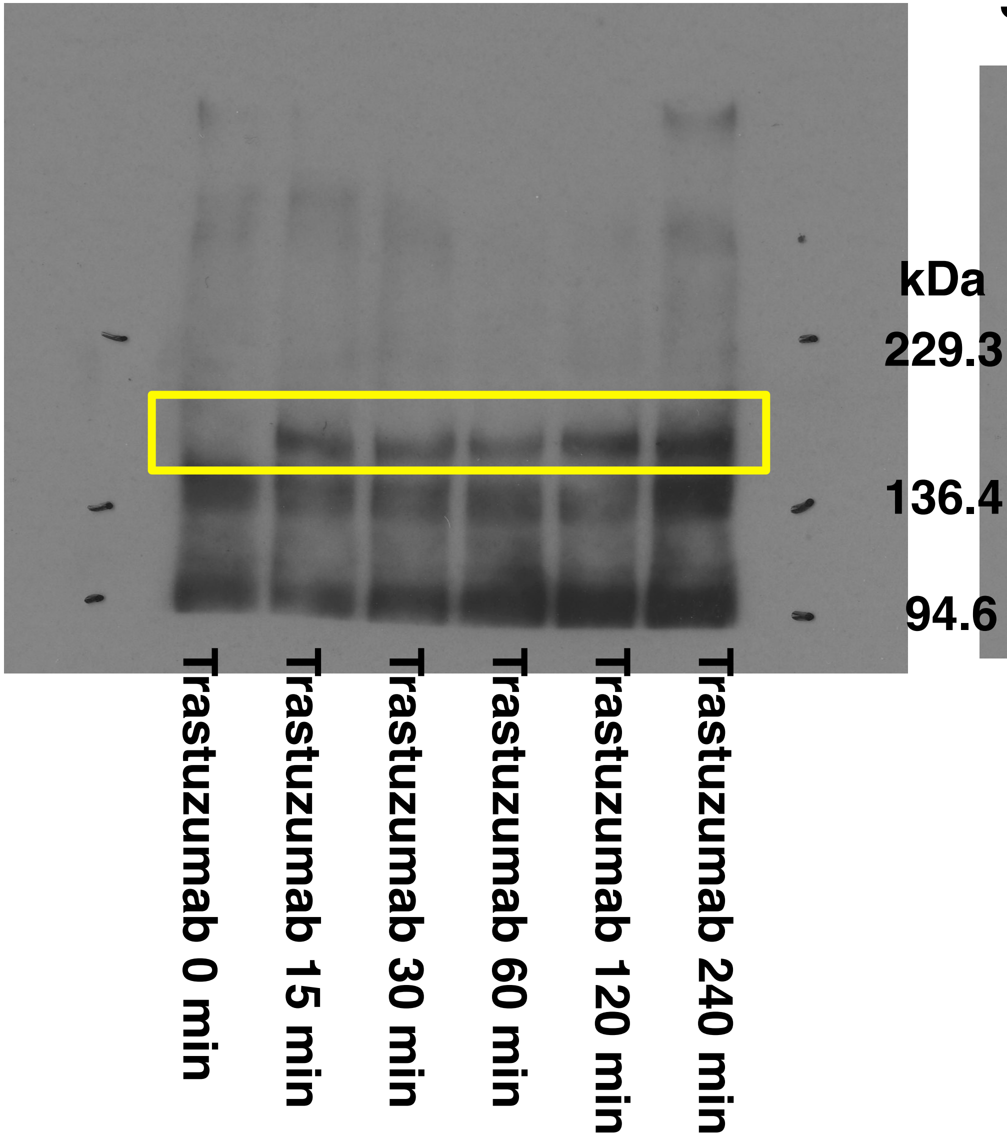

SBC-3/SN-38 IP: ubiquitin IB;HER2

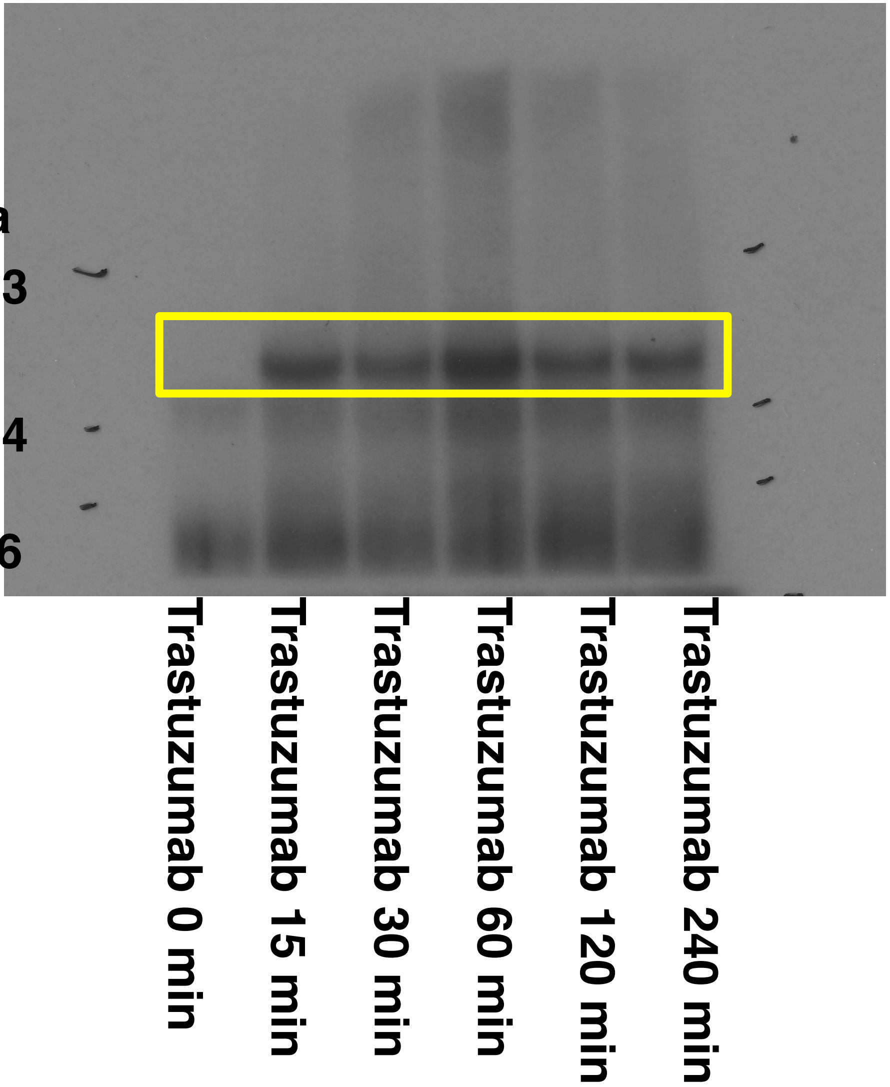

Supplement: Supplementary Information [file srep02669-s1.pdf]
